# Inhibition of TGF-β signalling in combination with nal-IRI plus 5-Fluorouracil/Leucovorin suppresses invasion and prolongs survival in pancreatic tumour mouse models

**DOI:** 10.1038/s41598-020-59893-5

**Published:** 2020-02-19

**Authors:** Eunji Hong, Sujin Park, Akira Ooshima, Chang Pyo Hong, Jinah Park, Jin Sun Heo, Siyoung Lee, Haein An, Jin Muk Kang, Seok Hee Park, Joon Oh Park, Seong-Jin Kim

**Affiliations:** 1 Precision Medicine Research Center, Advanced Institute of Convergence Technology, Seoul National University, Suwon, Gyeonggi-do 16229 Republic of Korea; 2 Department of Transdisciplinary Studies, Graduate School of Convergence Science and Technology, Seoul National University, Suwon, Gyeonggi-do 16229 Republic of Korea; 3 Department of Biological Science, Sungkyunkwan University, Suwon, 16419 Gyeonggi-do Republic of Korea; 4 TheragenEtex Bio Institute, Suwon, Gyeonggi-do 16229 Republic of Korea; 5 Department of Medicine, Samsung Medical Center, Sungkyunkwan University School of Medicine, Seoul, Republic of Korea; 6 Medpacto Inc., Seoul, Republic of Korea

**Keywords:** Chemotherapy, Tumour-suppressor proteins

## Abstract

Pancreatic ductal adenocarcinoma (PDAC) is one of the most aggressive malignancies. TGF-β is strongly expressed in both the epithelial and stromal compartments of PDAC, and dysregulation of TGF-β signalling is a frequent molecular disturbance in PDAC progression and metastasis. In this study, we investigated whether blockade of TGF-β signalling synergizes with nal-IRI/5-FU/LV, a chemotherapy regimen for malignant pancreatic cancer, in an orthotopic pancreatic tumour mouse model. Compared to nal-IRI/5-FU/LV treatment, combining nal-IRI/5-FU/LV with vactosertib, a TGF-β signalling inhibitor, significantly improved long-term survival rates and effectively suppressed invasion to surrounding tissues. Through RNA-sequencing analysis, we identified that the combination treatment results in robust abrogation of tumour-promoting gene signatures and positive enrichment of tumour-suppressing and apoptotic gene signatures. Particularly, the expression of tumour-suppressing gene *Ccdc80* was induced by vactosertib and further induced by vactosertib in combination with nal-IRI/5-FU/LV. Ectopic expression of CCDC80 suppressed migration and colony formation concomitant with decreased expression of epithelial-to-mesenchymal transition (EMT) markers in pancreatic cancer cells. Collectively, these results indicate that combination treatment of vactosertib with nal-IRI/5-FU/LV improves overall survival rates in a mouse model of pancreatic cancer by suppressing invasion through CCDC80. Therefore, combination therapy of nal-IRI/5-FU/LV with vactosertib could provide clinical benefits to pancreatic cancer patients.

## Introduction

Pancreatic cancer is one of the leading causes of cancer-related mortality in the world. The 5-year relative survival rate of pancreatic ductal adenocarcinoma (PDAC) patients was only 8% for all stages combined data in 2018^1^. Although 5-year relative survival rates in other cancer types have steadily increased over the years, improvement in pancreatic cancer is very slow because patients with pancreatic cancer are often diagnosed at advanced stages due to absence of noticeable symptoms in the early stages^2,3^. Moreover, control of pancreatic cancer is difficult owing to its aggressiveness, distal metastasis, resistance to most common treatments, and genetic and epigenetic alterations^4–6^.

The pancreatic cancer treatment paradigm has improved in the last 20 years. In clinical trials in the 1970s, 5-fluorouracil (5-FU) was used after resection^7^. Nowadays, the combination treatments such as FOLFIRINOX (oxaliplatin, irinotecan, fluorouracil (5-FU), and leucovorin (LV)) and gemcitabine plus *nab*-paclitaxel are the novel chemotherapy regimens used for patients with metastatic pancreatic adenocarcinoma (mPAC), displaying significantly better patient outcomes compared to the commonly used gemcitabine and providing the chance for salvage chemotherapy^8–10^. Nanoliposomal irinotecan (nal-IRI) has different pharmacokinetic properties from irinotecan owing to the outer PEGylated liposomes encapsulating the irinotecan sucrosofate salt. This very property of nal-IRI allows increased and prolonged levels of irinotecan and its metabolite SN-38 to carry out anti-tumour activity with low toxicity compared to free-irinotecan^11^. Based on the phase 2 clinical study of nal-IRI against pancreatic cancer^12^, the pivotal phase III NAPOLI-1 study has been conducted in gemcitabine-failed pancreatic cancer patients. In the NAPOLI-1 study, the combination of nal-IRI and 5-FU/LV significantly increased the median survival (6.1 months) compared to the 5-FU/LV control (4.2 months) or nal-IRI monotherapy (4.9 months)^13^. In this clinical trial, the effectiveness of nal-IRI is improved by co-treatment with 5-FU/LV rather than monotherapy of each regimen.

Regulation of TGF-β signalling is essential for controlling tumour progression and metastasis because TGF-β is involved in cell proliferation, epithelial-to-mesenchymal transition (EMT), angiogenesis, metastasis, and immune responses in cancer^14,15^. For example, when the TGF-βRI/II kinase inhibitor LY-2109761 was co-administered with gemcitabine in an orthotopic xenograft mouse model of pancreatic cancer, tumour growth and spontaneous metastasis were significantly reduced with improved median survival^16^. Loss of TGF-β signalling induced by SB-431542, an inhibitor of TGF-β type I receptor, increased sensitivity to rapamycin-induced apoptosis in MDA-MB-231-derived breast cancer^17^. Vactosertib (TEW-7197) is an orally bioavailable TGF-β signalling inhibitor that also targets the TGF-β type I receptor kinase^18,19^. Treatment of vactosertib reduces cancer cell migration, invasion, and metastasis of diverse cancers, including breast cancer, pancreatic cancer, and melanoma^20–22^. Recently, clinical studies of vactosertib-combined treatment with other anti-cancer drugs are ongoing in various cancer types. Particularly, vactosertib and FOLFOX combination therapy is undergoing evaluation for mPAC patients. Therefore, vactosertib could be an attractive candidate for pancreatic cancer therapy considering its ability to inhibit TGF-β signalling *in vivo*.

In this study, we investigated whether vactosertib manifests therapeutic benefits to pancreatic cancer patients by evaluating the effects of combination treatment of vactosertib with nal-IRI/5-FU/LV in an orthotopic mouse model of pancreatic cancer. We found that the combination dramatically increased the survival rate by suppressing invasion of pancreatic cancer cells. Through RNA-sequencing analysis, we found that genes involved in the regulation of the apoptotic process might be pertinent to the improved survival rate. Among these genes, we selected CCDC80, a tumour suppressor gene that was significantly induced by the combination treatment, to further investigate the underlying mechanism of the combination treatment. Indeed, ectopic expression of CCDC80 suppressed migration, colony formation, and expression of EMT markers. In summary, the combination of vactosertib with nal-IRI/5-FU/LV improved survival in the pancreatic cancer mouse model by inhibiting migration, invasion, and EMT of pancreatic cancer cells.

## Results

### Combination of vactosertib with nal-IRI/5-FU/LV significantly improved survival in a mouse pancreatic cancer model

**Figure 1:**
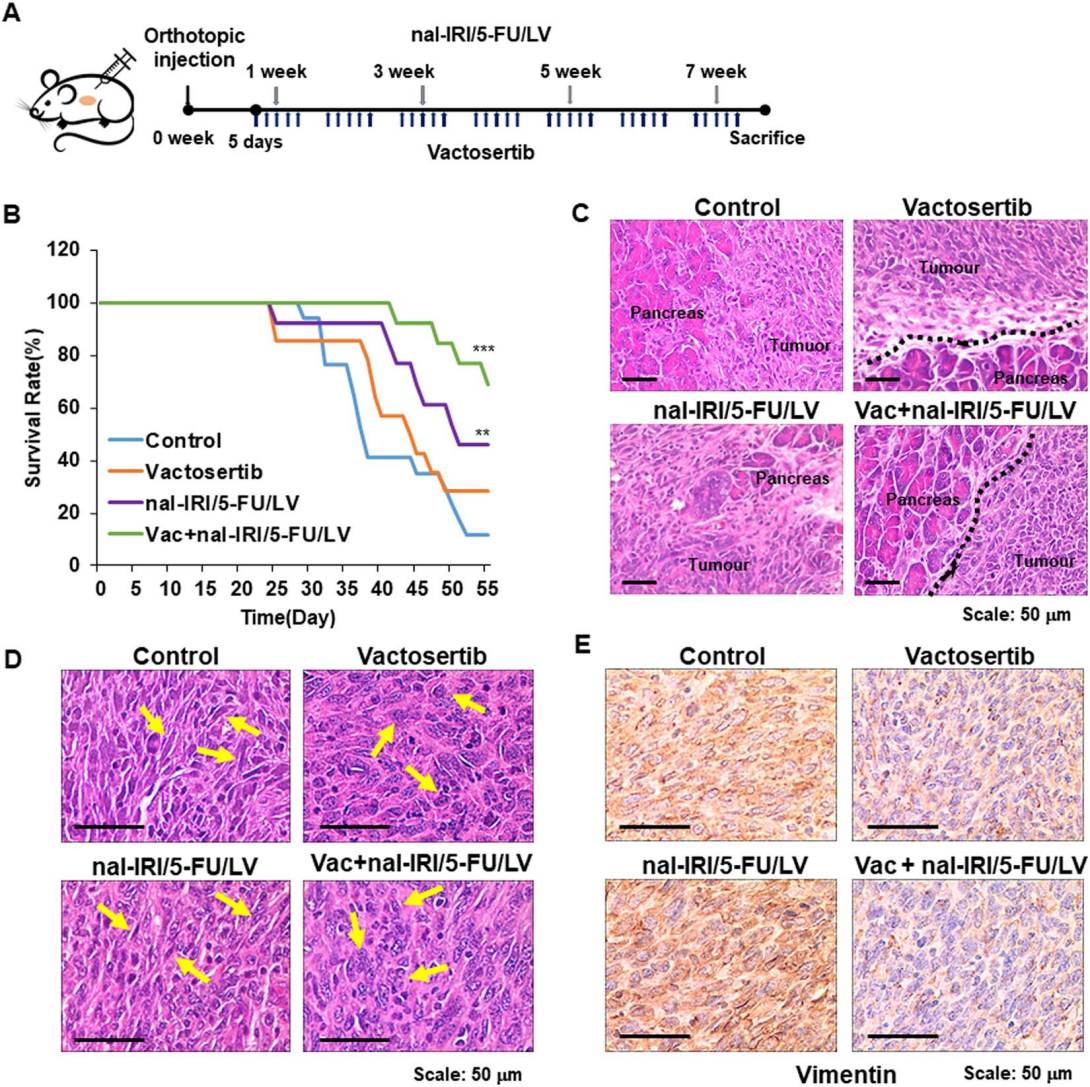
Survival improvement in response to combination treatment of vactosertib with nal-IRI/5-FU/LV in the orthotopic pancreatic cancer mouse model. (**A**) Experimental design of C57BL/6 syngeneic orthotopic mouse model using Panc02 murine pancreatic cancer cells. Five days after cell injection (3 × 10^6^), mice were randomized into 4 groups. Vactosertib was administered orally for 5 consecutive days followed by 2 days of resting period starting from day 6 post cell injection. nal-IRI/5-FU/LV was injected intraperitoneally every 2 weeks starting from day 8 post cell injection. (**B**) Relative survival rates of the control, vactosertib, nal-IRI/5-FU/LV, and combined treatment groups (***P < 0.0005 and **P < 0.005 compared to the control group) (**C**) Haematoxylin and eosin (H&E) staining of pancreatic tumour tissues showing tumour cell invasion to adjacent pancreas tissues. Black dotted lines indicate the borders between tumourous and normal pancreas without invading tumour cells. (**D**) The morphologies of tumour cells presented by H&E staining. Yellow arrows point out the representative mesenchymal (control and nal-IRI/5-FU/LV) and epithelial (vactosertib and Vac + nal-IRI/5-FU/LV) cell morphologies in the images. (**E**) The immunohistochemical staining of vimentin in tumour tissues of each group.

Prior to the investigation of the effects of combination of vactosertib with nal-IRI/5-FU/LV, we first examined whether blockade of TGF-β signalling through vactosertib, targeting TGF-β type I receptor, is beneficial for the treatment of pancreatic cancer by analysing the expression of *TGFBR1* in normal (n = 171) and pancreatic tumour (n = 179) tissues using TCGA database. We found that *TGFBR1* was significantly overexpressed in tumour tissues (Supplementary Fig. 1A). We then tested the effect of vactosertib on PANC-1 human pancreatic cancer cell. As shown in Supplementary Fig. 1B,C, vactosertib treatment restored TGF-β1-mediated suppression of *CDH1* and reduced TGF-β1-mediated increase of *CDH2*, *SNAI1*, and *SNAI2*, ultimately suppressing TGF-β1-induced migration of PANC-1 cells. Based on these observations, we investigated whether vactosertib co-administration enhances the anti-tumour effects of nal-IRI/5-FU/LV in an orthotopic pancreatic cancer mouse model using murine Panc02 pancreatic cancer cells (Fig. 1A). We observed that both nal-IRI/5-FU/LV and Vac + nal-IRI/5-FU/LV effectively suppressed tumour growth (Supplementary Fig. 2A). Both vactosertib and nal-IRI/5-FU/LV monotherapy enhanced survival, but the survival rate was significantly improved by more than 60% in the Vac + nal-IRI/5-FU/LV-administered group (Fig. 1B). Interestingly, as demonstrated by H&E staining, borders between tumour tissues and pancreatic parenchyma were clearly demarcated in the vactosertib alone- and Vac + nal-IRI/5-FU/LV-administered groups (Fig. 1C). Indeed, cancer cells in the vactosertib- and Vac + nal-IRI/5-FU/LV-administered groups showed epithelial phenotype, while cells in the control and nal-IRI/5-FU/LV-administered groups exhibited mesenchymal phenotype with a spindle-like shape (Fig. 1D). Immunohistochemical analysis also demonstrated that the expression of vimentin, a representative EMT marker, was markedly reduced in both vactosertib alone- and Vac + nal-IRI/5-FU/LV-administered groups (Fig. 1E). These results suggest that both vactosertib and its combination with nal-IRI/5-FU/LV might reduce invasiveness and EMT of pancreatic cancer. Furthermore, we observed that administration of nal-IRI/5-FU/LV alone could induce fibrotic changes such as accumulation of α-smooth muscle actin (α-SMA) and deposition of collagen in the pancreatic tumour tissues that might interfere with the function of pancreatic parenchyma adjacent to cancer (Supplementary Fig. 2B). However, combination treatment of vactosertib with nal-IRI/5-FU/LV significantly suppressed fibrotic changes in the pancreatic tumour tissues by reducing α-SMA expression and collagen deposition. Taken together, combination treatment of vactosertib with nal-RI/5-FU/LV demonstrated favourable therapeutic effects on the syngeneic mouse model of pancreatic cancer through reduction of primary tumour sizes and suppression of invasion to surrounding tissues.

In addition, we examined whether combination of vactosertib with nal-IRI monotherapy increases survival rates, even though nal-IRI monotherapy showed lower efficiency than nal-RI/5-FU/LV in the clinical trial^13^. Compared to either nal-IRI or vactosertib monotherapy, combination of vactosertib with nal-IRI resulted in a significantly prolonged survival rate in the mouse model of pancreatic cancer (Supplementary Fig. 3A,B). Similar to Vac + nal-IRI/5-FU/LV-administered mice, histological analysis showed increased epithelial phenotype and reduced tumour cell invasion in vactosertib-administered groups (Supplementary Fig. 3C), suggesting that the combination of vactosertib with nal-IRI also improves survival rates and reduces invasiveness of pancreatic cancer in the mouse model.

### Co-treatment of vactosertib with nal-IRI/5-FU suppresses migration, invasion, and EMT of pancreatic cancer cells

**Figure 2:**
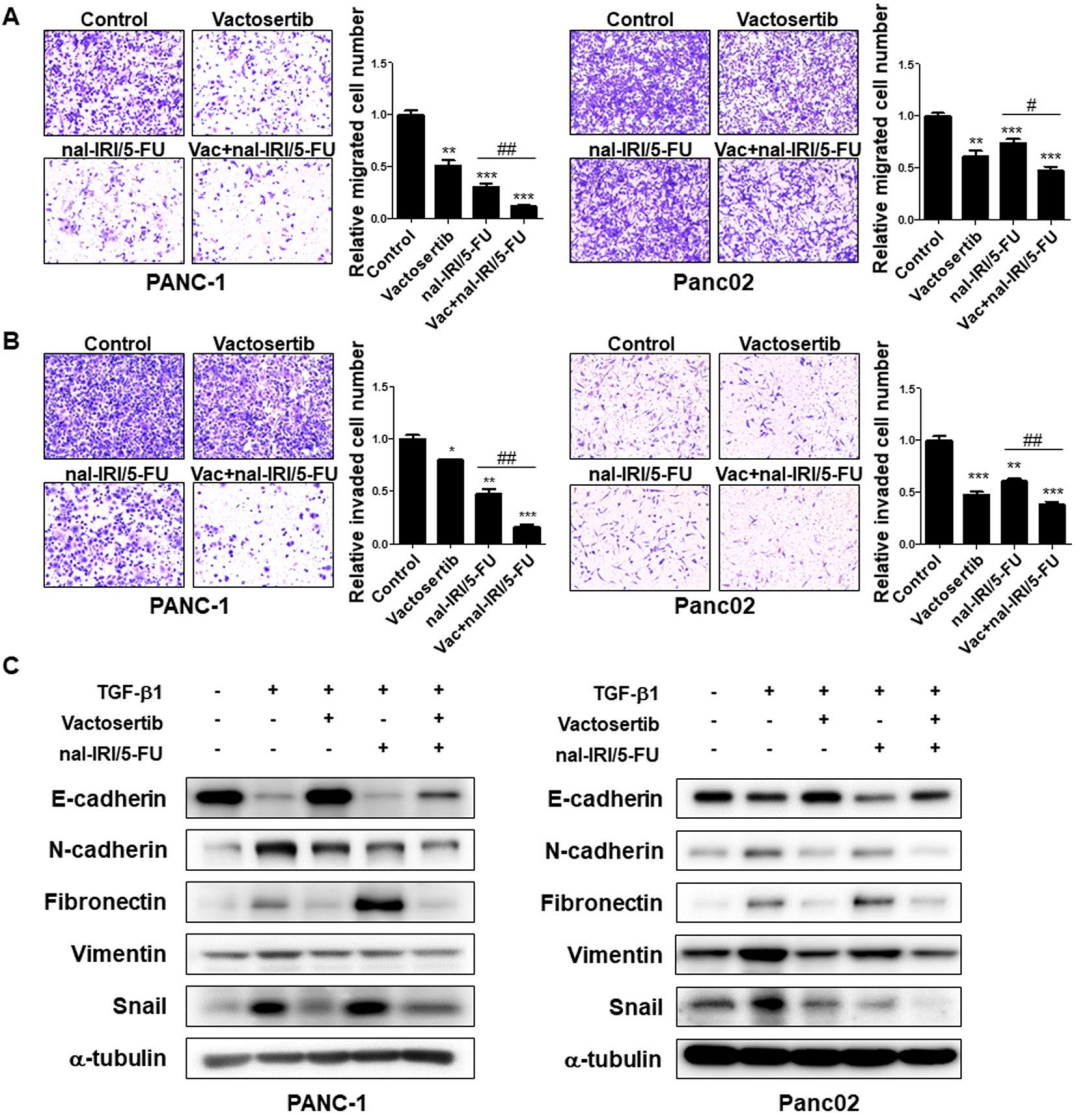
Synergistic effect of vactosertib with nal-IRI/5-FU on migration, invasion, and EMT of pancreatic cancer cells. (**A**) Transwell cell migration assay. Cells were treated with the indicated reagent(s) and incubated for 48 hours before placed in a migration chamber. 1 × 10^5^ of PANC-1 (left) or 5 × 10^4^ of Panc02 (right) cells were then seeded in migration chambers for 48 hours. The relative number of migrated cells in each group was counted. (**B**) Cell invasion assay. Cells were treated with the indicated reagent(s) and incubated for 48 hours before placed in an invasion chamber. 2 × 10^5^ PANC-1 (left) or 1 × 10^5^ Panc02 (right) cells were then seeded in invasion chambers for 48 hours. The number of invaded cells in each group was counted. Note that the combination of vactosertib with nal-IRI/5-FU inhibits migration and invasion in PANC-1 and Panc02 pancreatic cancer cells. (**A,B**) The values for migrated/invaded number of cells represent the mean ± SD of triplicate data. ***P < 0.0005, **P < 0.005, and *P < 0.05 compared to the control group; ^##^P < 0.005 and ^#^P < 0.05 compared to the nal-IRI-treated group. (**C**) Western blot analysis for EMT markers. Vactosertib was treated for 2 hours prior to incubation with nal-IRI/5-FU in the presence of 5 ng/ml TGF-β for 48 hours. The blots are cropped and the full-length images are presented in Supplementary Fig. 8. Note that vactosertib and its combination with nal-IRI/5-FU restore the TGF-β-mediated reduction of E-cadherin and the increase of mesenchymal markers.

Based on *in vivo* observations, we next examined the combined effect of vactosertib with nal-IRI/5-FU in pancreatic cell lines. Because leucovorin (LV) is used as an adjuvant chemical to reduce the toxicity of anti-cancer drugs *in vivo*, we only used 5-FU for *in vitro* experiments. In addition to Panc02 cells that were used for *in vivo* experiments, we selected PANC-1 and SNU2491 human pancreatic cancer cell lines that showed responses to TGF-β1 for further investigation (Supplementary Fig. 4). In order to examine the effect of vactosertib on invasiveness of pancreatic cancer cells *in vitro*, we first performed migration and invasion assays. Each vactosertib and nal-IRI/5-FU treatment significantly reduced migration of PANC-1, Panc02, and SNU2491 cells while the combination treatment of vactosertib with nal-IRI/5-FU reduced migration even further (Fig. 2A and Supplementary Fig. 5A). The synergistic effect of vactosertib and nal-IRI/5-FU was further investigated with invasion assay using a chamber coated with matrigel that mimics extracellular matrix. Compared to the pancreatic cancer cells of the control, vactosertib, and nal-IRI/5-FU groups that had extendedly degraded matrigel and migrated through the chamber, the combination treatment reduced invasive capability of pancreatic cancer cells (Fig. 2B and Supplementary Fig. 5B). We also studied the effect of vactosertib and its combination with nal-IRI/5-FU by examining EMT responses. First of all, PANC-1, Panc02, and SNU2491 cells all showed TGF-β1-induced EMT showing reduced expression of epithelial marker E-cadherin as well as increased expression of mesenchymal markers such as N-cadherin, Fibronectin, and Snail. Compared to nal-IRI/5-FU, vactosertib effectively reduced the TGF-β1-mediated induction of mesenchymal marker expression and restored the TGF-β1-mediated reduction of E-cadherin expression. However, combination treatment of vactosertib with nal-IRI/5-FU significantly ameliorated TGF-β1-induced EMT of these pancreatic cancer cells (Fig. 2C and Supplementary Fig. 5C). These data suggest that when combined with nal-IRI/5-FU, vactosertib exerts synergistic effects on suppressing migration and invasion of pancreatic cancer cell lines by regulating EMT.

### Transcriptome profiling of pancreatic tumour tissues following combination treatment of vactosertib with nal-IRI/5-FU/LV

**Figure 3:**
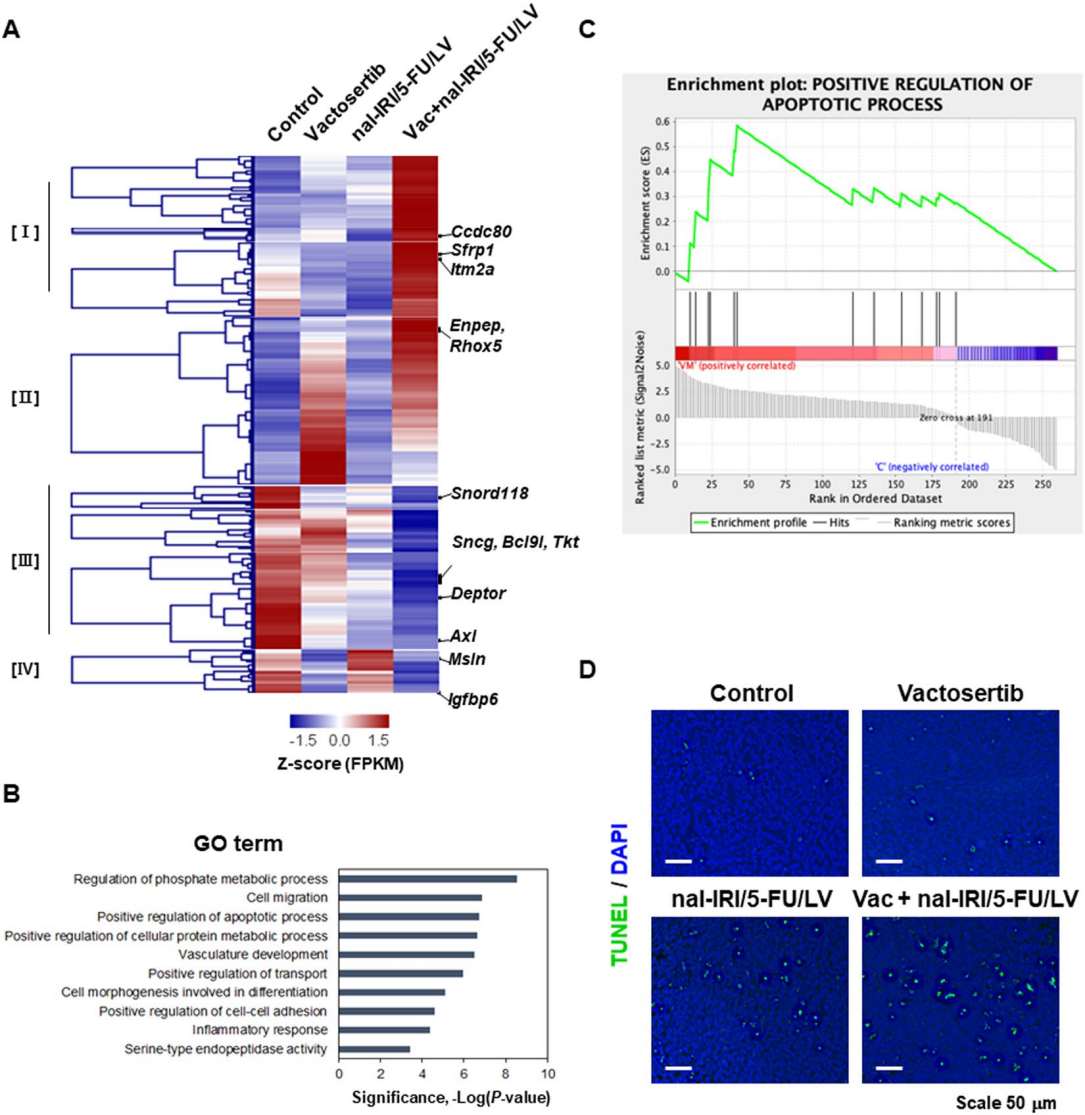
RNA-sequencing analysis of pancreatic tumour tissues obtained from orthotopic mouse model administered with vactosertib, nal-IRI/5-FU/LV, and the combination. (**A**) Heatmap plot of DEGs using the hierarchical clustering method. (**B**) Top 10 pathways characterized by from GO enrichment analysis of DEGs (cutoff of P < 0.001). (**C**) GO enrichment plot for positive regulation of apoptotic process plotted by Gene Set Enrichment Analysis (GSEA). (**D**) TUNEL assay of pancreatic tumour tissues used for RNA sequencing. Note that more apoptotic cells were shown in the tumour tissues obtained from mice administered with combination of vactosertib with nal-IRI/5-FU/LV.

To further explore the mechanism by which combination treatment of vactosertib with nal-IRI/5-FU/LV has on the survival of the pancreatic tumour mouse model, we performed RNA-seq analysis of the pancreatic tumours. The sequencing analysis yielded an average of 89.7 (±25.9) million reads per library (Additional file 1). Compared to the control tumours, we identified 571, 648, and 976 differentially expressed genes (DEGs) in the pancreatic tumours that had been administered with vactosertib, nal-IRI/5-FU/LV, and the combination treatment, respectively. Among the DEGs, we identified a total of 325 genes (cluster II, III, and IV) that were differentially expressed in response to either vactosertib or nal-IRI/5-FU/LV monotherapy (Fig. 3A). Interestingly, these genes were significantly regulated by the combination treatment and exhibited a reverse expression pattern between the control and the Vac + nal-IRI/5-FU/LV-administered groups. To gain a better understanding of the involvement of these genes in the effects of combination treatment in pancreatic cancer, functional categories of the genes were subjected to gene ontology (GO) enrichment analysis. Our analysis of the genes revealed significant functions associated with regulation of phosphate metabolic processes (*P* = 3 × 10^−9^), cell migration (*P* = 1.3 × 10^−7^), positive regulation of apoptotic processes (*P* = 1.8 × 10^−7^), vasculature development (*P* = 3 × 10^−7^), and positive regulation of transport (*P* = 1.1 × 10^−7^) (Fig. 3B, Supplementary Table 1). In particular, enrichment of the genes such as *Sfrp1, Sfrp2, Il1b, Lpar1, Pmaip1, Ctla4, Cyp1b1, Map3k5, Nfatc4, Aldh1a2, Nr4a1, Clip3*, and *Dusp1* in the functional category of positive regulation of apoptotic process was supported by gene set enrichment analysis (GSEA) (Fig. 3C). These findings suggest that the combination treatment might exert an effect on the apoptotic process of pancreatic cancer. To confirm whether the combination treatment regulates apoptosis, we performed TUNEL assay using tumour tissue sections. As shown in Fig. 3D, the apoptotic cells were significantly increased in Vac + nal-IRI/5-FU/LV-administered tumour tissues. Taken together, our results indicate that combination treatment of vactosertib with nal-IRI/5-FU/LV might reduce pancreatic tumour progression not only by suppressing invasion, but also by enhancing apoptosis while simultaneously regulating DEGs.

### Identification of *Ccdc80* and tumorigenesis-related genes regulated by combination treatment of vactosertib with nal-IRI/5-FU/LV

**Figure 4:**
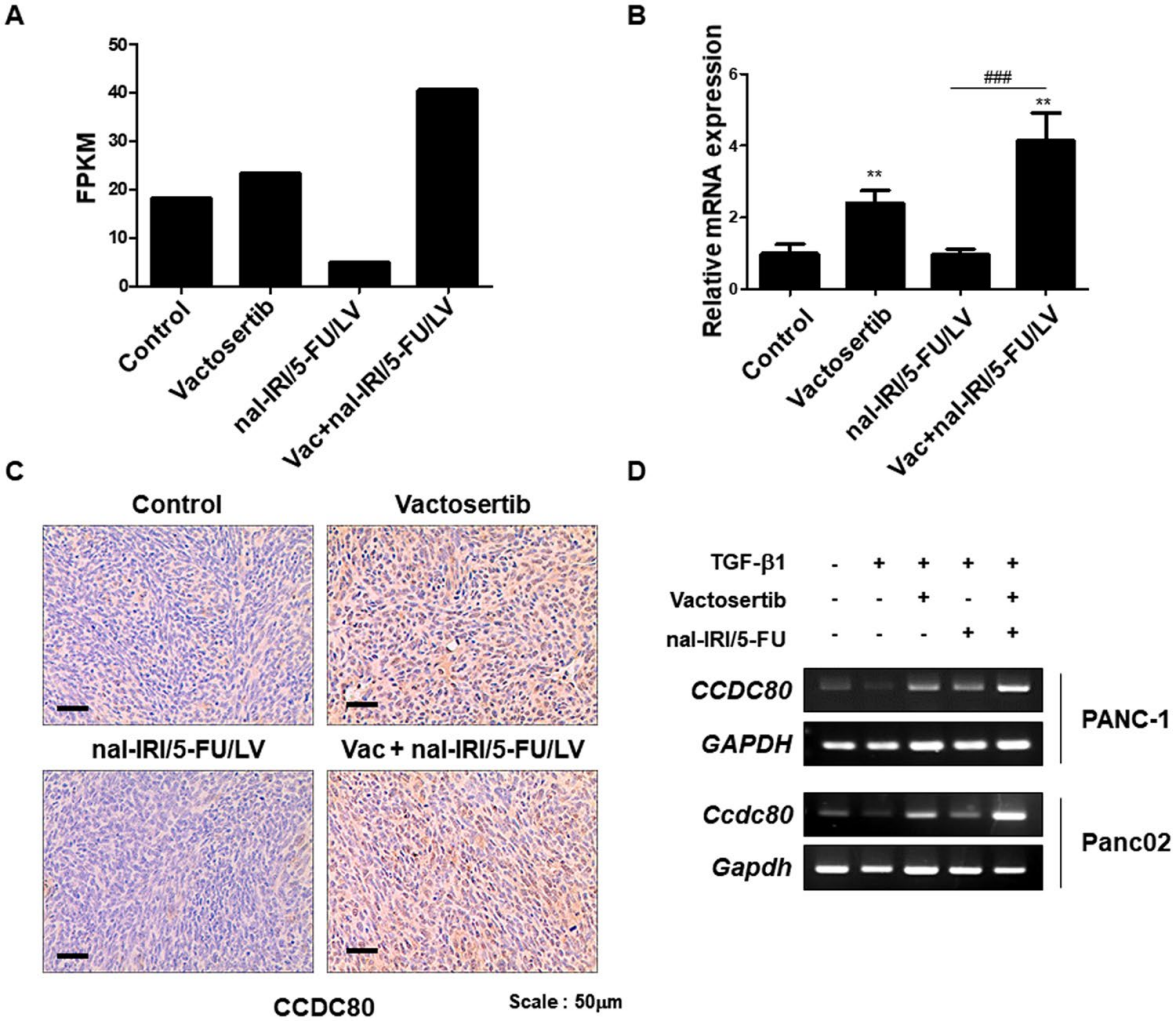
Up-regulation of *CCDC80* expression by vactosertib and its combination with nal-IRI/5-FU/LV. (**A**) FPKM value of *Ccdc80* in the tumour tissues from RNA sequencing. (**B**) qRT-PCR result validating *Ccdc80* mRNA expression in mouse tumour tissues. The values represent the mean ± SD of triplicate samples. **P < 0.005 compared to the control group. ###P < 0.0005 compared to the nal-IRI/5-FU/LV group. (**C**) Immunohistochemical staining of mouse tumour tissues with CCDC80. Note that CCDC80 expression is increased in the tumour tissues from the mice administered with vactosertib or its combination with nal-IRI/5-FU/LV. (**D**) RT-PCR analysis revealing up-regulation of CCDC80 by vactosertib and its combination with nal-IRI/5-FU in PANC-1 and Panc02 cell lines.

From RNA-Seq analysis, we found that tumour suppressors such as *Ccdc80*, *Itm2a*, and *Sfrp1* were significantly up-regulated by the combination treatment of vactosertib with nal-IRI/5-FU/LV compared to the control group and the nal-IRI/5-FU/LV group (Fig. 4A and Supplementary Fig. 6A). We further confirmed altered expression levels of the selected genes by quantitative RT-PCR (Fig. 4B and Supplementary Fig. 6B). We found that the expression levels of several oncogenes, including *Axl*, *Tkt*, *Sncg*, and *Bcl9l*, were down-regulated by the treatment of vactosertib with nal-IRI/5-FU/LV (Supplementary Fig. 6A,B). Consistently, the expression levels of the tumour suppressor genes were higher in the healthy mouse pancreatic tissues than in the pancreatic tumour tissues obtained from the orthotopic mouse model using Panc02 cells. On the other hand, the expression levels of the oncogenes were higher in the tumour tissues than in the healthy tissues (Supplementary Fig. 6C). Among the genes identified, *Ccdc80* is one of the known tumour suppressors regulating the expression of E-cadherin^23^. According to previous studies, CCDC80 is related to cancer cell motility in thyroid, colorectal, and pancreatic cancers^24,25^. Down-regulation of *Ccdc80* is detected in pancreatic cancer cell lines and primary tumours, and ectopic expression of CCDC80 promotes cell growth inhibition and sensitizes cells to apoptosis in colon and pancreatic cancer cell lines^25^. Interestingly, *Ccdc80* was induced by vactosertib, and it was further induced by the combination treatment of vactosertib with nal-IRI/5-FU/LV. Through immunohistochemical staining of Ccdc80, we confirmed that the protein expression of Ccdc80 was significantly increased in the cancer tissues obtained from the vactosertib and Vac + nal-IRI/5-FU/LV-administered mice compared to the ones from the control and nal-IRI/5-FU/LV-administered mice (Fig. 4C). We also analysed mRNA levels of *CCDC80* in the vactosertib and nal-IRI/5-FU-treated PANC-1 and Panc02 cell lines. Since the expression of *CCDC80* was suppressed by TGF-β1, inhibition of TGF-β signalling by vactosertib significantly induced *CCDC80* expression, which was further induced by vactosertib in combination with nal-IRI/5-FU (Fig. 4D). These results suggest that CCDC80 might be an important mediator for combination treatment of vactosertib with nal-IRI/5-FU/LV in reducing pancreatic tumour progression.

### Ectopic expression of CCDC80 in pancreatic cancer cells decreases migration, colony formation, and EMT

**Figure 5:**
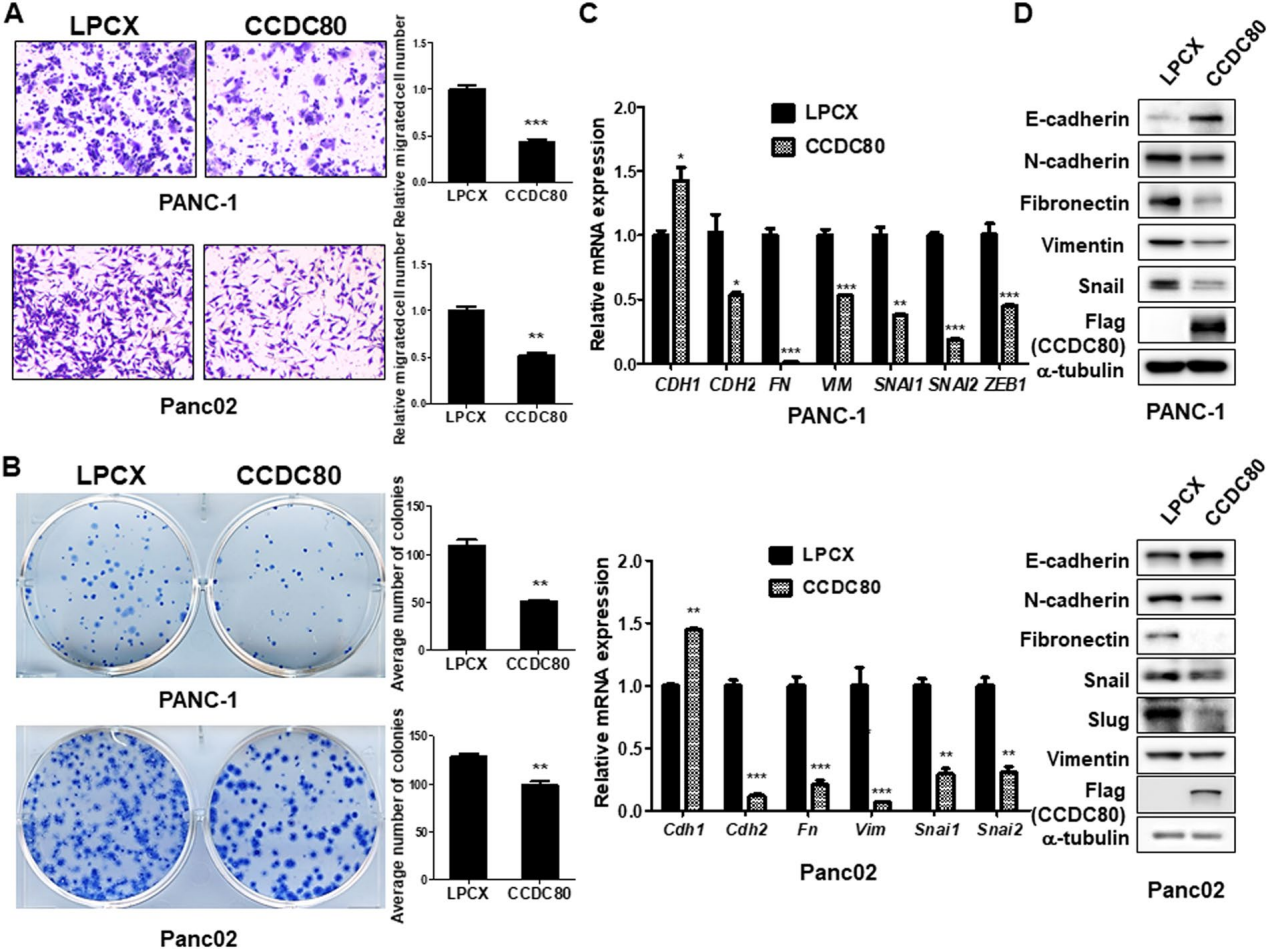
Ectopic expression of CCDC80 reducing migration, colony formation, and EMT in pancreatic cancer cells. (**A**) Transwell migration assay measuring migration abilities of PANC-1 and Panc02 cells expressing LPCX or CCDC80. (**B**) Representative images of colonies stained with methylene blue in PANC-1 and Panc02 cells stably expressing CCDC80. (**C**) qRT-PCR results showing down-regulation of EMT marker expression in PANC-1 and Panc02 cells by ectopic expression of CCDC80. All the data is represented as the mean of three repeated values. ***P < 0.0005, **P < 0.005, and *P < 0.05 compared to the control. (**D**) Western blot analysis showing reduction of EMT markers in PANC-1 and Panc02 with stably expressing CCDC80. The blots are cropped, and the original blots are presented in Supplementary Fig. 8. Note that overexpression of CCDC80 decreases EMT marker expression in pancreatic cancer cells.

We further investigated whether CCDC80 plays a role in EMT of pancreatic cancer cells. Consistent with down-regulation of *Ccdc80* in the pancreatic tumour tissues, mRNA expression of *CCDC80* was significantly decreased in most of the pancreatic cancer cell lines compared to HPDE normal pancreatic cell line (Supplementary Fig. 6C,D). To investigate the role of CCDC80 in pancreatic cancer progression, we generated PANC-1 and Panc02 cells stably expressing 3Flag-CCDC80. We found that the ectopic expression of CCDC80 significantly reduced cell migration and colony formation concomitant with down-regulation of mesenchymal markers such as *CDH2*, *FN*, *VIM*, *SNAI1*, *SNAI2*, and *ZEB1*, and up-regulation of epithelial marker *CDH1* in both PANC-1 and Panc02 cells (Fig. 5A–C). Western blot analysis demonstrated that the expression level of E-cadherin was increased and the expression levels of mesenchymal markers such as N-cadherin, Fibronectin, Vimentin, Snail, and Slug were significantly decreased by ectopic expression of CCDC80 (Fig. 5D). The same results were also observed in SNU2491 cells stably expressing CCDC80 (Supplementary Fig. 7). Collectively, these findings suggest that CCDC80 might participate in the underlying mechanism of combination treatment of vactosertib with nal-IRI/5-FU/LV in suppressing invasiveness and subsequent pancreatic cancer progression.

## Discussion

TGF-β signalling plays a vital role in pancreatic cancer initiation and progression^26^. At least one mutation in the TGF-β downstream genes is detected in most pancreatic cancer patients^27^. Inactivation of a TGF-β signalling mediator leads to a loss of tumour suppressive role of TGF-β in pancreatic cancer^28,29^. TGF-β plays a dual role during tumorigenesis. It suppresses tumour progression by promoting cell cycle arrest and apoptosis in early-stage of pancreatic carcinogenesis. On the other hand, it functions as a tumour promoter by activating the Smad4-independent signalling pathway, promoting cell motility, invasion, EMT, and metastasis and decreasing anti-tumour immune responses in advanced stages^30^. Thus, inhibition of TGF-β signalling in the late stages of cancer progression could be an effective strategy for treating pancreatic cancer. In this study, we found that treatment of vactosertib, a TGF-β signalling inhibitor, with either nal-IRI/5-FU/LV or nal-IRI significantly improved overall survival in a mouse model of pancreatic cancer by suppressing EMT and invasion of pancreatic cancer cells. In addition, tumour microenvironment, such as cancer-associated fibroblasts, plays a crucial role in metastasis^31^. We observed that treatment of vactosertib and its combination with nal-IRI/5-FU/LV effectively reduced α-SMA and collagen deposition in the pancreatic tumour tissues, whereas nal-IRI/5FU/LV treatment induced fibrotic changes that might lead to pancreas dysfunction. Considering the involvement of TGF-β in regulating tumour microenvironment, inhibition of TGF-β through vactosertib might suppress fibrotic changes during tumourigenesis. Taken together, our results suggest that combination treatment of vactosertib with other anti-cancer drugs might be useful in treating advanced-stage pancreatic cancer.

Through RNA-seq analysis, the DEGs related to TGF-β signalling were identified in response to combination treatment of vactosertib with nal-IRI/5-FU/LV. For example, BCL9L and SNCG are involved in TGF-β-induced EMT, migration, invasion, and metastasis^32,33^. The expression of BCL9L is up-regulated in pancreatic cancer^34^, and knockdown of BCL9L diminishes TGF-β-induced EMT responses in pancreatic cancer cells and inhibits liver metastasis *in vivo*^32^. BCL9L expression level determines the aggressiveness of pancreatic tumours, presenting lower expression in SPN, a benign pancreatic tumour, than in PDAC, an aggressive type^34^. The expression of SNCG is up-regulated in breast, colon, and pancreatic cancers^35–37^. SNCG is involved in perineural invasion associated with local recurrence and dismal prognosis in relation with TGF-β or Twist in pancreatic cancer^33^. In this study, we found that combination treatment of vactosertib with nal-IRI/5-FU/LV significantly reduced *SNCG* and *BCL9L* expression. Moreover, combination treatment of vactosertib with nal-IRI/5-FU/LV induced well-known tumour suppressors ITM2A and SFRP1. In ovarian cancer, expression level of ITM2A is reduced, and the disease-free survival rate is prolonged in patients in the ITM2A-high patient group^38^. Loss of SFRP1 has been detected in hepatocellular carcinoma, colon cancer, breast cancer, and many other cancers associated with poor prognosis^39–42^. Even though we focused on CCDC80 in the present study, it would be worth investigating whether ITM2A and SFRP1 are involved in the action of combination treatment of vactosertib with nal-IRI/5-FU/LV in ameliorating pancreatic cancer.

According to previous studies, CCDC80 (Dro1) is a secreted protein induced by oestrogen^43^. It plays a role in osteoblast and adipocyte differentiation, embryonic skeletal development, and glucose homeostasis^44,45^. In regard to cancer, mRNA expression of CCDC80 is often down-regulated in various cancer cell lines and tumour tissues, suggesting the tumour-suppressive role of CCDC80^24,43,46,47^. Overexpression of CCDC80 decreases clonogenic capacities of cancer cells, including PANC-1 and BxPC-3 pancreatic cancer cells, and increases CD95-induced apoptosis^25^. CCDC80 is also known to mediate signals through the FAK/E-cadherin axis, which regulates cell migration^23^. In addition to these roles of CCDC80, we revealed that the ectopic expression of CCDC80 could also suppress cell migration, colony formation, and EMT in PANC-1, Panc02, and SNU2491 pancreatic cancer cells.

In conclusion, this study demonstrates that combination treatment of vactosertib with nal-IRI/5-FU/LV improves pancreatic cancer survival by suppressing cell migration, invasion, and EMT, highlighting a potential clinical application of this combination therapy for PDAC patients.

## Materials and Methods

### Cell lines

The human pancreatic cancer cell line PANC-1 was obtained from American Type Culture Collection (ATCC, VA, USA), and the C57BL/6 syngeneic pancreatic adenocarcinoma cell line Panc02 was provided from Medpacto (Korea). Both cell lines were maintained in DMEM with high glucose (WelGENE), supplemented with 10% FBS (WelGENE), 100 U/ml penicillin and 100 μg/ml streptomycin.

### Reagents

Vactosertib was obtained from Medpacto (Korea) and solubilized in gastric fluid. Nanoliposomal irinotecan (Onivyde) was a kind gift from Prof. Joon Oh Park from Samsung Seoul Hospital, and 5-fluorouracil was purchased from MCE (Cat. No. HY-90006/CS-0993) and leucovorin (calcium folinate) from TCI (Cat. No. C2235). Both 5-FU and LV were solubilized in PBS. Antibodies against E-cadherin (Cat. No. 610181), N-cadherin (Cat. No. 610921), and Fibronectin (Cat. No. 610077) were obtained from BD Biosciences. Antibodies against Vimentin (Abcam, Cat. No. ab9978), Snail (Cell Signaling, Cat. No. 3879), Slug (Santa Cruz, Cat. No. sc-15391), and CCDC80 (Bioss, Cat. No. bs-7992R) were diluted in 3% BSA solution. Anti-mouse and anti-rabbit secondary antibodies were purchased from Millipore.

### TCGA analysis

TCGA analysis for TGFBR1 expression data was obtained from the GEPIA database in human normal pancreatic tissues (N = 171) and pancreatic cancer tissues (N = 179) (http://gepia.cancer-pku.cn). The P value was calculated by unpaired two-tailed Student’s t-tests.

### Syngeneic murine model of pancreas orthotopic tumour injection

Six-week-old male immunocompetent C57BL/6 mice were purchased from Japan SLC, Inc. We injected 3 × 10^6^ Panc02 cells with a 1:1 ratio of Matrigel (Corning, Cat. No. 356231) into the tail of pancreas. Five days after implantation, mice were randomized into four treatment groups: control, vactosertib, nal-IRI/5-FU/LV, and combination of vactosertib and nal-IRI/5-FU/LV (n = 10–15 per group). The mice were treated with 25 mg/kg vactosertib in gastric fluid by oral gavage five times a week and/or 10 mg/kg nal-IRI, 25 mg/kg 5-FU, and 45 mg/kg leucovorin via intraperitoneal (i.p.) injection once in two weeks. The control group was injected with gastric fluid (orally) and PBS (i.p.) at the same time. We followed up on the body weight to detect the health of mice and tracked the death of mice for survival. We collected tumour samples from each group at the same time. All animal experiments were approved by the Woojung Bio Animal facility (Suwon, Korea) and all methods were performed in accordance with the relevant guidelines and regulations.

### Tumour tissue fixation and immunohistochemistry

Pancreatic tumour tissues were fixed in 10% neutral buffered formalin overnight and then embedded in paraffin blocks. Slides were stained with haematoxylin and eosin (H&E) and Masson’s trichrome. The antibodies used for immunohistochemistry (IHC) were α-SMA (Novus, Cat. No. NBP1-30894), vimentin (Abcam, Cat. No. ab92547) and CCDC80 (Bioss, Cat. No. bs-7992R).

### TUNEL assay

TUNEL assay to stain the apoptotic cells of the mouse pancreatic cancer tissues was performed using DeadEnd Fluorometric TUNEL system (Promega. Cat. No. G3250) with the provided protocol. In brief, the tumour tissue sections were deparaffinised with xylene and rehydrate using graded ethanol. To permeabilize the tissues, the tissue slides were incubated with Proteinase K and then covered with equilibration buffer and rTdT buffer. SSC buffer was used to stop the reaction, and the nuclei were stained with DAPI (Vector lab, Cat. No. H-1200). Apoptotic cells (green) were detected with fluorescence microscope (100×).

### Migration and invasion assays

Either vactosertib, nal-IRI/5-FU, or combination of vactosertib and nal-IRI/5-FU was added to 5 × 10^5^ pancreatic cancer cell lines on 60 mm plates for 48 hours. Cells were detached from the plates by trypsin-EDTA (WelGENE). Approximately 5 × 10^4^ cells were seeded in transwells for migration (Falcon, Cat. No. 353097) and invasion (Corning, Cat. No. 354480) assays and incubated for 48 hours in a 37 °C cell culture incubator. Then, we fixed the migrated and invaded cells with 70% ethanol and stained them with 0.05% crystal violet.

### Colony formation assay

We seeded 5 × 10^2^ Panc02 cells per well on a 6-well plate and incubated the plate for one week with medium changes every three days. After the cells formed colonies, we fixed the cells and stained them with methylene blue in 50% ethanol.

### Western blot analysis

We followed the western blot protocol as described previously^48^. Briefly, PANC-1 and Panc02 cells were washed twice with PBS and lysed with RIPA buffer, 50 mM Tris-HCl (pH 7.5), 150 mM NaCl, 1% Nonidet P-40, 0.5% sodium deoxycholate, 0.1% sodium dodecyl sulphate and a protease inhibitor cocktail (Sigma, P8340) on ice for 30 minutes. We measured protein concentrations using a Pierce BCA protein assay kit (Thermo Scientific, Cat. No. 23225). Proteins were heated at 95 °C in SDS sample buffer, separated by SDS-PAGE, transferred to PVDF membrane and incubated overnight with the indicated primary antibodies. The blots were detected by Amersham Imager 600 system (GE Healthcare Life Sciences, UK). The blots are cropped, and the full-length images are included in Supplementary Information.

### RNA isolation, reverse transcription, and quantitative RT-PCR

RNA from cell lines and murine pancreatic tumour tissues was isolated using an easy-BLUE Total RNA extraction kit (Promega, Cat. No. 17061). Two micrograms of RNA were used for reverse transcription by M-MLV reverse transcriptase (Promega, Cat. No. M1705). Quantitative RT-PCR was performed using a QuantStudio5 Real-Time PCR instrument (Applied Biosystems) with TOPreal qPCR 2x PreMIX (Enzynomics, Cat. No. RT500M). Gene expression was normalized to *Gapdh*.

### RNA sequencing and data analysis

The quality of purified mouse tumour RNA was measured using an Agilent 2100 Bioanalyzer (Agilent Technologies, CA). All extractions delivered an RNA integrity number (RIN) value of 9.2–9.9 and 28 S/18 S ratio of 1.1–1.6. RNA-Seq libraries were prepared using a TruSeq RNA Sample Prep Kit according to the manufacturer’s manual (Illumina, Inc., San Diego, CA). Using 1 μg of the qualified RNA in each sample, poly(A) mRNA was enriched by magnetic beads with oligo(dT) and then fragmented into approximately 200 bp inserts by sonication. The synthesized cDNA was subjected to end-repair and poly(A) tailing and connected with sequencing adapters using a TruSeq Stranded mRNA Sample Prep Kit (Illumina, Inc., San Diego, CA). The proper cDNA fragments, purified by a BluePippin instrument (Sage Science, MA) according to the manufacturer’s instructions, were selected for further PCR amplification. The final library sizes ranged between 350–450 bp. Subsequently, the libraries were subjected to paired-end sequencing with a 100 bp read length using an Illumina HiSeq 2500 platform. The reads generated by the Illumina HiSeq 2500 platform were then processed for quality with the following criteria: low-quality reads that contained more than 10% inaccurate bases were marked as ‘N,’ and reads with >40% low quality bases (i.e., quality score < 20) were discarded. The quality scores were analysed using the fastqc tool (https://www.bioinformatics.babraham.ac.uk/projects/fastqc/), and the filtration process was performed using in-house scripts.

### Bioinformatics analysis

Clean reads were aligned to the mouse reference genome (Ensembl 84) using TopHat^49^ with a set of its gene model annotations. Gene expression was quantified using Cufflinks^50^. Differential expression (DE) analysis between sample groups of interest was performed using Cuffdiff^50^ with cut-offs set at P* < *0.05 and ≥1.5-fold change. In addition, because of possible noise at very low levels of expression, genes expressed at ≥1 FPKM in at least one sample were retained for DE analysis. Hierarchical clustering for selected genes was conducted with MeV (http://mev.tm4.org) using the Euclidean distance and complete linkage method. Functional annotation for module members was performed using DAVID^51^, and relevant gene ontology (GO) terms were selected with a cut-off of P* < *0.001. *Gene set enrichment analysis* (*GSEA*) was further performed to examine the critical GOs of the transcriptome^52^. For this, the estimated expression levels of all genes were run through GSEA, and then the enrichment scores were calculated according to the rank-ordered gene list. Significance scores were computed by 1,000 nonparametric permutation tests.

### Statistics

All the data were expressed as the mean with SD. Statistical significance between all quantitative data are analyzed by Student *t*-tests and one-way ANOVA using GraphPad Prism version 5 (GraphPad Software Inc.).

## Supplementary information

Supplementary information.

Supplementary Table S1

## Data Availability

The RNA-Seq data is available in the NCBI Sequence Read Archive (SRA) database under the accession number SRP229044 (https://trace.ncbi.nlm.nih.gov/Traces/study/?acc=SRP229044&o=acc_s%3Aa).
