# Supplementary material for: Inhibition of TGF-β signalling in combination with nal-IRI plus 5-Fluorouracil/Leucovorin suppresses invasion and prolongs survival in pancreatic tumour mouse models
Source: Sci Rep. 2020 Feb 19;10:2935. doi: 10.1038/s41598-020-59893-5 (PMC7031242; doi:10.1038/s41598-020-59893-5)
Supplement: Supplementary file 1 — Supplementary information. [file 41598_2020_59893_MOESM1_ESM.pdf]

Title: Inhibition of TGF-β signalling in combination with nal-IRI plus 5-Fluorouracil/Leucovorin suppresses invasion and prolongs survival in pancreatic tumour mouse models

Eunji Hong, Sujin Park, Akira Ooshima, Chang Pyo Hong, Jinah Park, Jin Sun Heo, Siyoung Lee, Haein An, Jin Muk Kang, Seok Hee Park, Joon Oh Park, and Seong-Jin Kim

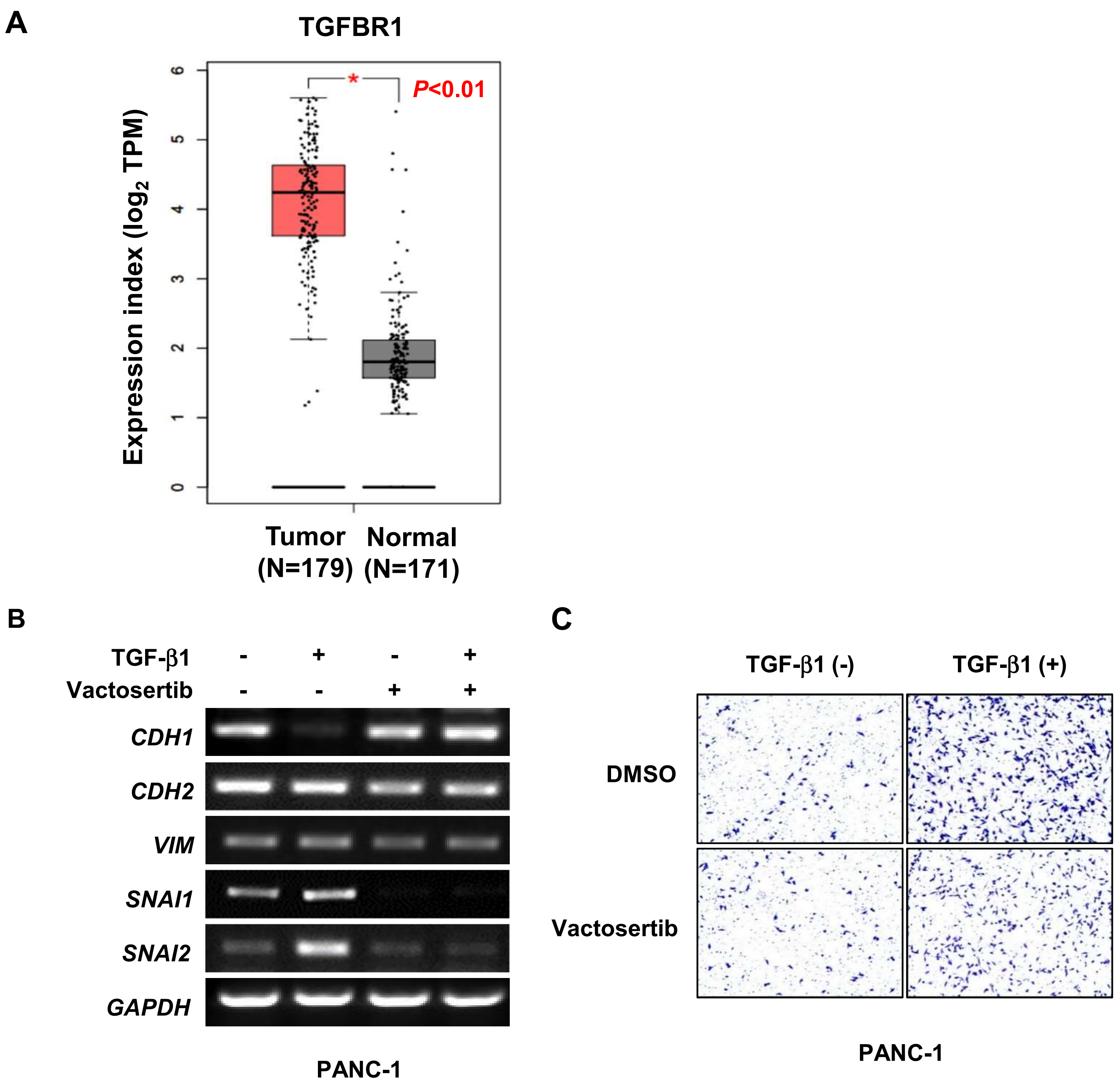

Supplementary figure 1. Overexpressed TGFBR1 in human pancreatic tumour and inhibition of the TGF-β1-induced migration and EMT response by vactosertib in pancreatic cancer cell

(A) TCGA analysis of TGFBR1 expression in pancreatic cancer patient tissue. Tumour (red box) has overexpressed level of TGFBR1 compared to normal tissue (grey box). (B) RT-PCR showing EMT marker expression and (C) migration assay of PANC-1 cells treated with vactosertib and TGF-β1. For *in vitro* experiments, PANC-1 cells were pre-treated with vactosertib for 2 hours and incubated with TGF-β1 for 48 hours.

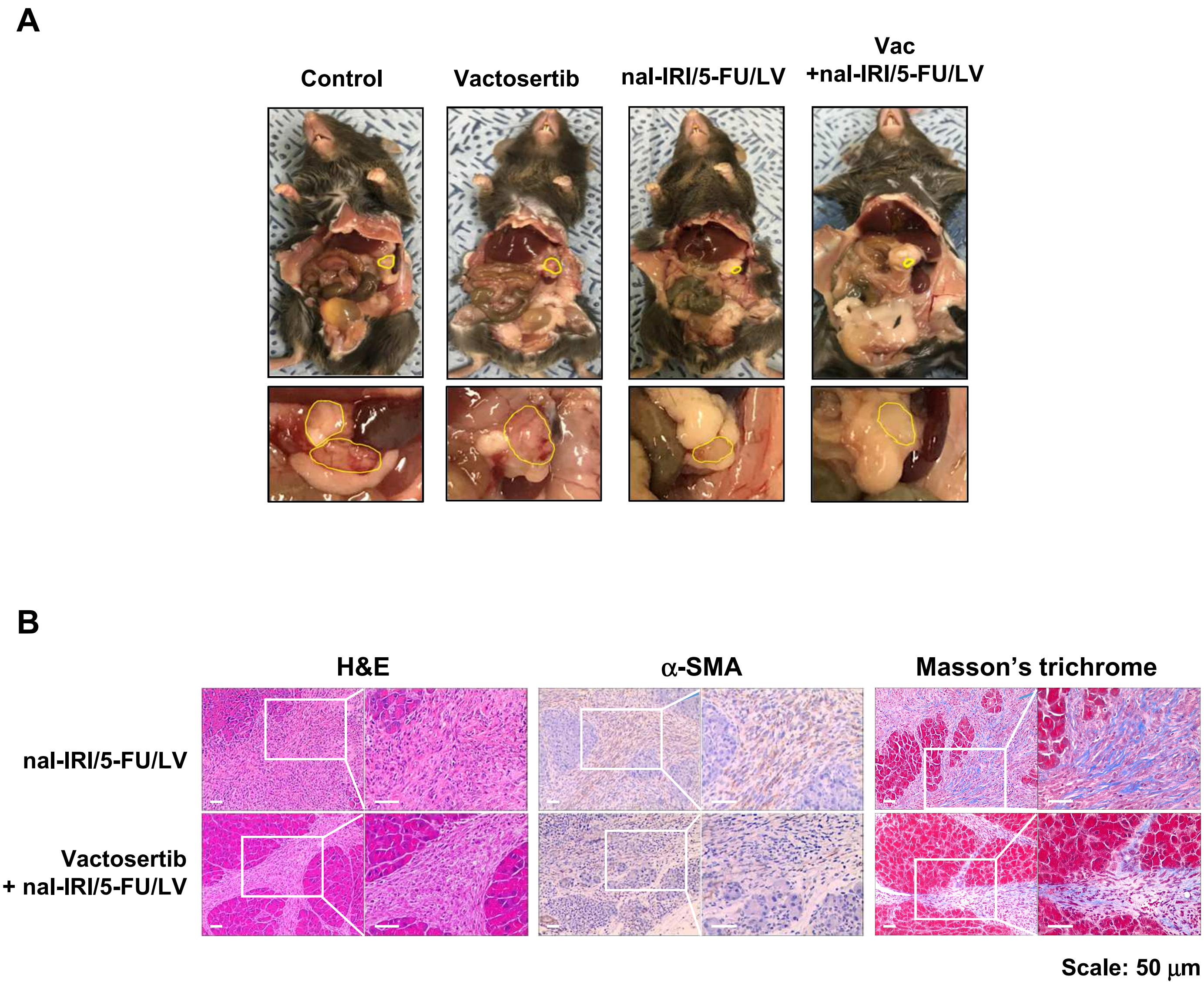

Supplementary figure 2. Suppression of tumour growth and fibrosis of pancreas by combination of vactosertib and nal-IRI/5-FU/LV

(A) Representative pictures of mice in each group (above) and enlarged pictures of the pancreatic tumour tissues outlined by yellow lines (bottom). The reduction in tumour size by combination of vactosertib with nal-IRI/5-FU/LV was detected. (B) Fibrotic changes occur in nal-IRI/5-FU/LV and its combination with vactosertib. H&E staining (left), immunohistochemistry of α-SMA (middle), and Masson's trichrome staining (right, collagen in blue) of the pancreas and tumour tissues.

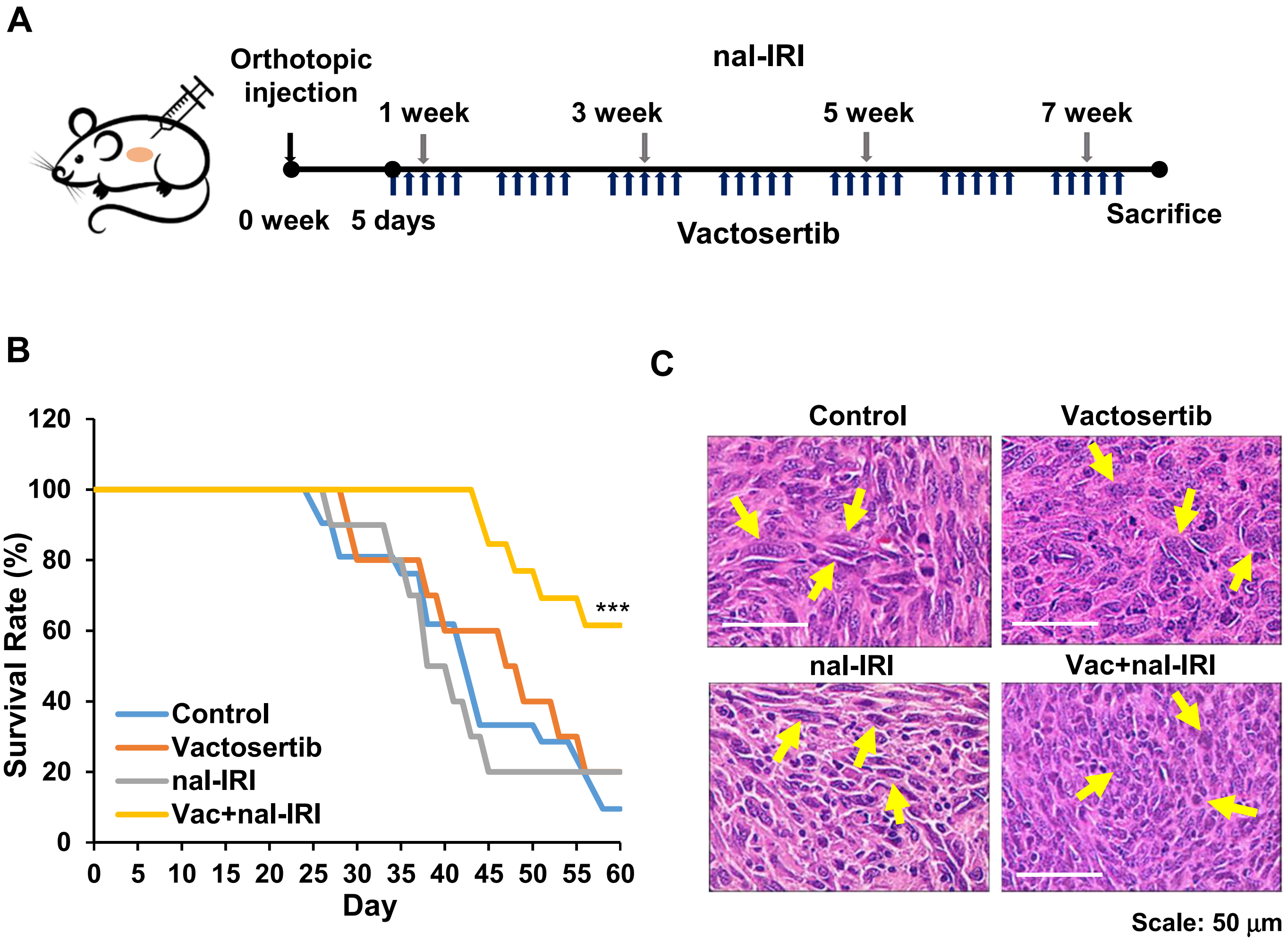

Supplementary figure 3. Effect of combination treatment of vactosertib with nal-IRI on survival rates in the pancreatic tumour mouse model

(A) Scheme for combination treatment with vactosertib and nal-IRI in a mouse model. The experimental design is shown in Figure 1. (B) Overall survival of each group (***P<0.0005 control;Vac+nal-IRI). (C) H&E staining to compare the cancer cell morphology of mouse tumour tissues. Mesenchymal (control and nal-IRI) and epithelial (vactosertib and Vac+nal-IRI) cell type morphologies are pointed out by yellow arrows.

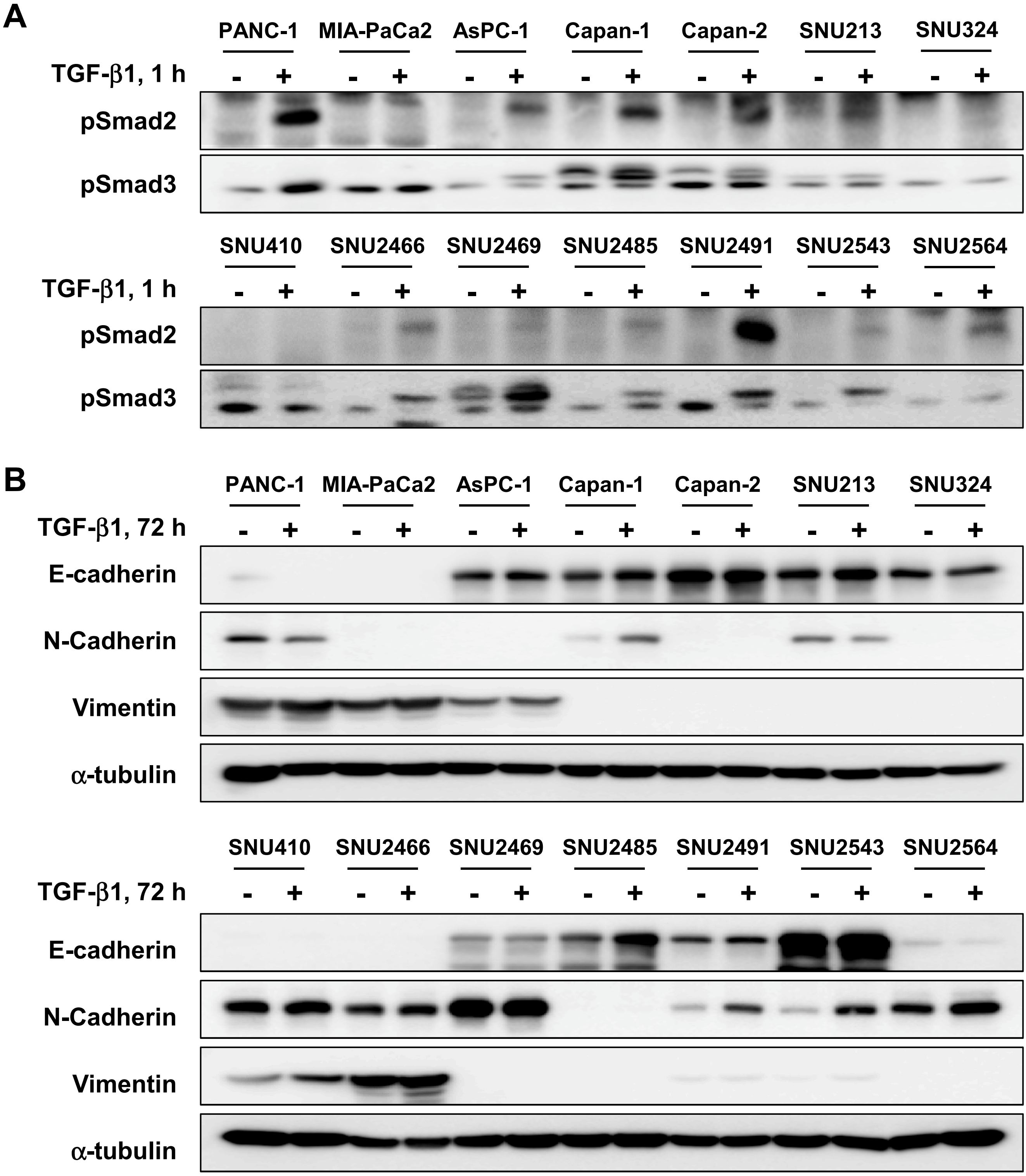

Supplementary figure 4. TGF-β responses in PANC-1 and SNU2491 cells among 14 human pancreatic cancer cell lines

(A) Western blot analysis showing increased phosphorylation of Smad2 and Smad3 by TGF-β1 (5 ng/ml, 1 h) and (B) induction of EMT marker expression by TGF-β1 treatment (5 ng/ml, 72 h) in 14 human pancreatic cancer cell lines. The blots are cropped, and the full-length images are shown in Supplementary Figure 8.

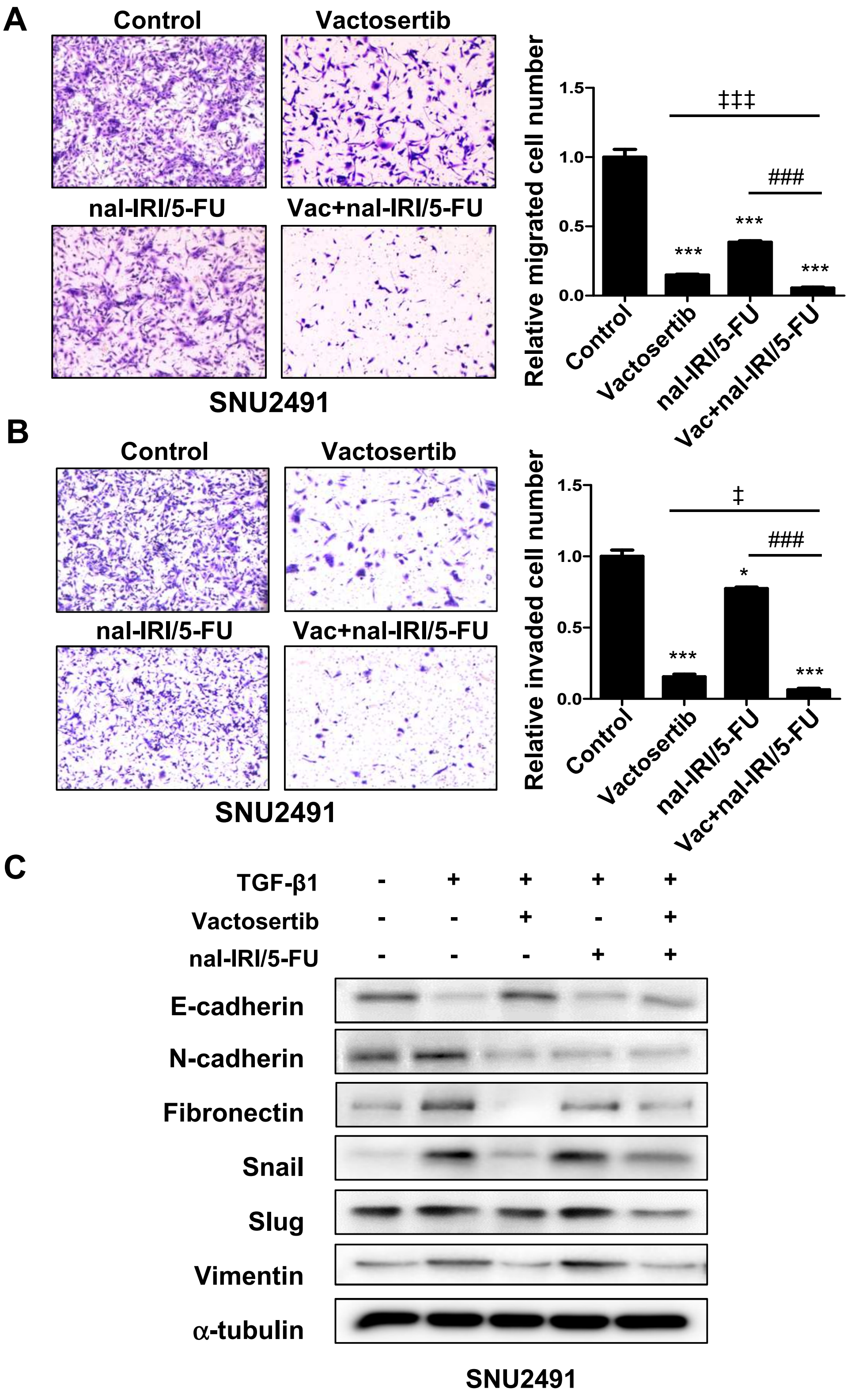

Supplementary figure 5. Reduction in migration/invasion ability and EMT response by combination treatment of vactosertib with nal-IRI/5-FU in SNU2491

(A) Migration assay and (B) invasion assay. (C) Western blot analysis for EMT markers. The blots are cropped, and the full-length images are presented in Supplementary Figure 8. The values represent the mean ± SD of triplicate data. ***P<0.0005 and *P<0.05 compared to the control group; ‡‡‡P<0.0005 and ‡P<0.05 compared to the vactosertib group; ###P<0.0005 compared to the nal-IRI/5-FU group.

GO term	-Log(P-value)	P-value	Genes	Count
**Regulation of phosphate metabolic process**	8.517222213	3.04E-09	S100A8, S100A9, TLR2, GJA1, LPAR1, PMAIP1, ENPEP, TGFB2, BDNF, MAP3K5, TNFRSF11B, GPC3, SERPINE2, PPP1R1B, IL1B, CEP85, ICOSL, C5AR2, PTGER3, NGP, CAMP, ELANE, CCNL1, INHBB, BTG2, CD80, CCR2, CLIP3, HBB-BT, PTAFR, NKD1, NKD2, CYP1B1, PODN, TNFRSF25, ENPP2, C3, MMD, 1500015O10RIK, CCL8, HSPA1B, BDKRB2, RPL38, ITM2A, CALCA, RASGRP1, FNDC1, TNFRSF19, AGRN, HAP1, MDFI, PLAT, HAVCR2, SMAD7, NR4A1, PBP2, DOCK7, SAMSN1, RPS5, CISH, ATF3, SFRP1, DUSP1, SFRP2, FABP4, INS2, GFRA2, VLDLR	68
**Positive regulation of apoptotic process**	6.734165339	1.84E-07	STEAP3, CYP1B1, S100A8, CTRB1, CD248, S100A9, CTLA4, NR4A1, NR4A3, PMAIP1, LPAR1, TGFB2, INHBB, ALDH1A2, MAP3K5, BDNF, ATF3, SFRP1, DUSP1, SFRP2, FNDC1, IL1B, BIK, NFATC4, AGRN, CLIP3	26
**Vasculature development**	6.519784325	3.02E-07	CYP1B1, ENPP2, C3, COL3A1, GJA1, ENPEP, TGFB2, ALDH1A2, GPC3, ZFP950, HEY1, IL1B, NFATC4, COL8A1, ANGPTL4, COL4A1, PTPRM, NGP, SMAD7, PDPN, CAMP, CCDC80, NR4A1, SFRP2, CCR2, COL1A2, FOXC1, MYLK	28
**Positive regulation of cellular protein metabolic process**	6.636239391	2.31E-07	NKD1, NKD2, CYP1B1, S100A8, ENPP2, TNFRSF25, C3, S100A9, MMD, TLR2, CCL8, GJA1, LPAR1, PMAIP1, TGFB2, CALCA, TNFRSF11B, BDNF, MAP3K5, GPC3, RASGRP1, FNDC1, IL1B, TNFRSF19, AGRN, MDFI, HAVCR2, C5AR2, ICOSL, PTGER3, SMAD7, CAMP, ELANE, CCNL1, DOCK7, INHBB, CD80, SFRP2, CCR2, CLIP3, INS2, PTAFR, GFRA2, VLDLR	44
**Cell migration**	6.881851014	1.31E-07	PODN, CYP1B1, S100A8, ENPP2, CD248, S100A9, COL3A1, TLR2, CCL8, GJA1, POSTN, LPAR1, ENPEP, MDK, SDC2, TGFB2, CALCA, ZFP950, SEMA3G, IL1B, DCLK1, PLAT, C5AR2, PTGER3, PTPRM, PDPN, ELANE, NR4A1, DOCK7, CDKL5, RUFY3, TNS1, SFRP1, SFRP2, CXCL13, CCR2, FOXC1, STC1, MYLK, PTAFR	40
**Positive regulation of transport**	5.946103178	1.13E-06	RNASEL, S100A8, C3, S100A9, TLR2, GJA1, POSTN, PMAIP1, TGFB2, GPC3, RASGRP1, IL1B, TMEM30B, PTX3, KCNQ1, HAP1, HAVCR2, LPL, CHP2, MCU, NR4A3, ABCG1, RUFY3, INHBB, TULP1, SYNE1, CTSK, SFRP2, STC1, CLIP3, INS2, PROS1, MYLK, PTAFR	34
**Cell morphogenesis involved in differentiation**	5.115372754	7.67E-06	ENPP2, GJA1, POSTN, SDC2, TGFB2, BDNF, HEY1, SEMA3G, NFATC4, AGRN, DCLK1, ISLR2, ACTB, NGEF, PTPRM, SMAD7, DOCK7, NR4A3, CDKL5, RUFY3, SLIT3, SYNE1, SFRP1, SFRP2, MFAP2, STC1, CDH11, VLDLR	28
**Positive regulation of cell-cell adhesion**	4.57618754	2.65E-05	HAVCR2, CD83, ICOSL, CD80, SMAD7, CXCL13, RASGRP1, CCR2, ELANE, IL1B, NR4A3, IGFBP2, PTAFR	13
**Inflammatory response**	4.373837458	4.23E-05	HAVCR2, C5AR2, S100A8, TNFRSF25, C3, C4B, S100A9, TLR2, CCL8, CCRL2, CALCA, TNFRSF11B, CXCL13, CCR2, IL1B, PTAFR	16
**Serine-type endopeptidase activity**	3.416043399	3.84E-04	PLAT, CFB, CTRB1, ELANE, PAMR1, MCPT2, HP, CELA2A, CFD, HGFAC, PRSS35	11

Supplementary table 1. Gene list of GO enrichment

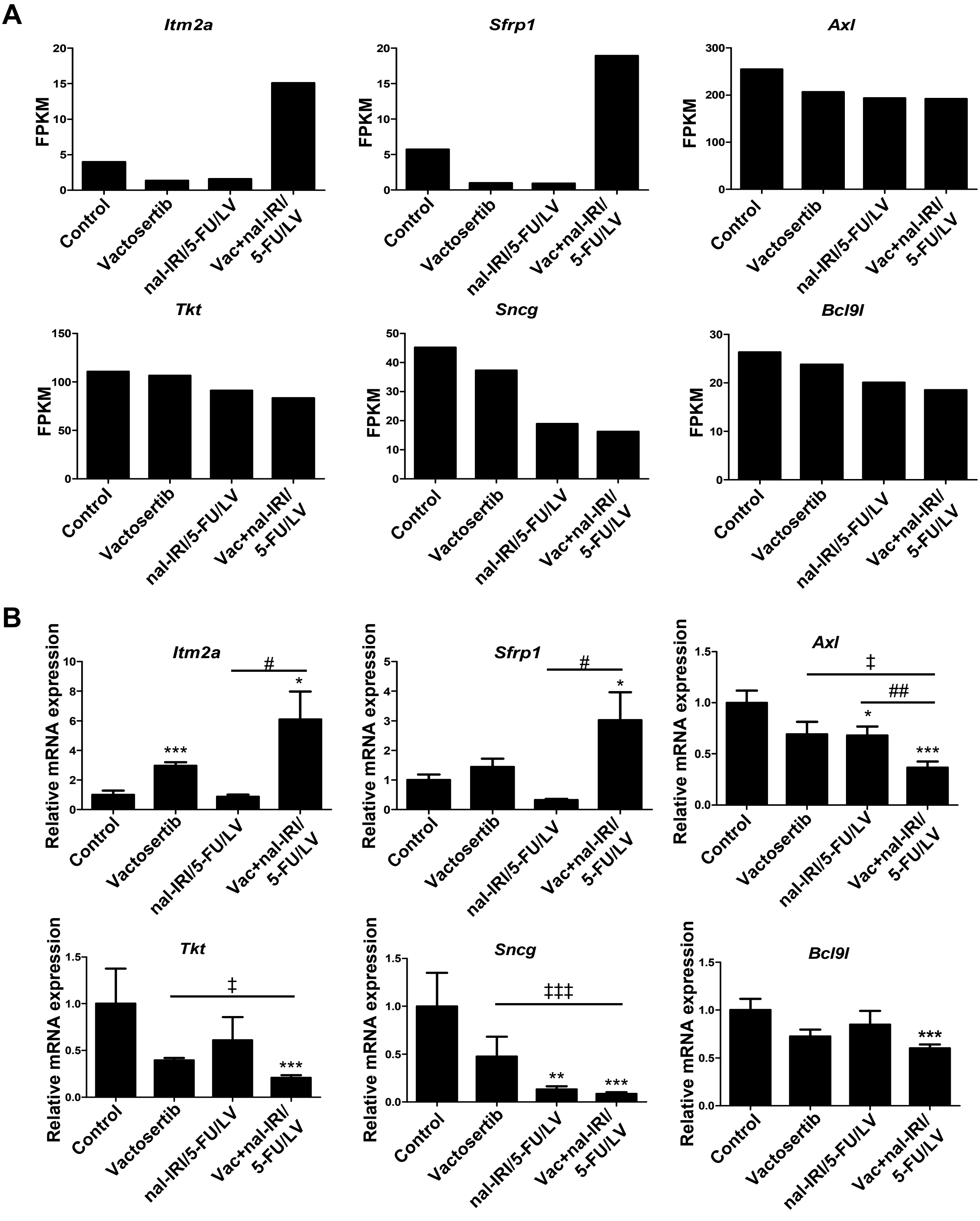

Supplementary figure 6. FPKM values and validation of target genes from RNA sequencing

(A) FPKM values of representative DEGs from RNA sequencing. (B) Relative mRNA expression in the mouse tumour ***P<0.0005, **P<0.005, and *P<0.05 compared to the control group; ‡‡‡P<0.0005 and ‡P<0.05 compared to the vactosertib group; ##P<0.005 and #P<0.05 compared to the nal-IRI/5-FU/LV group.

C

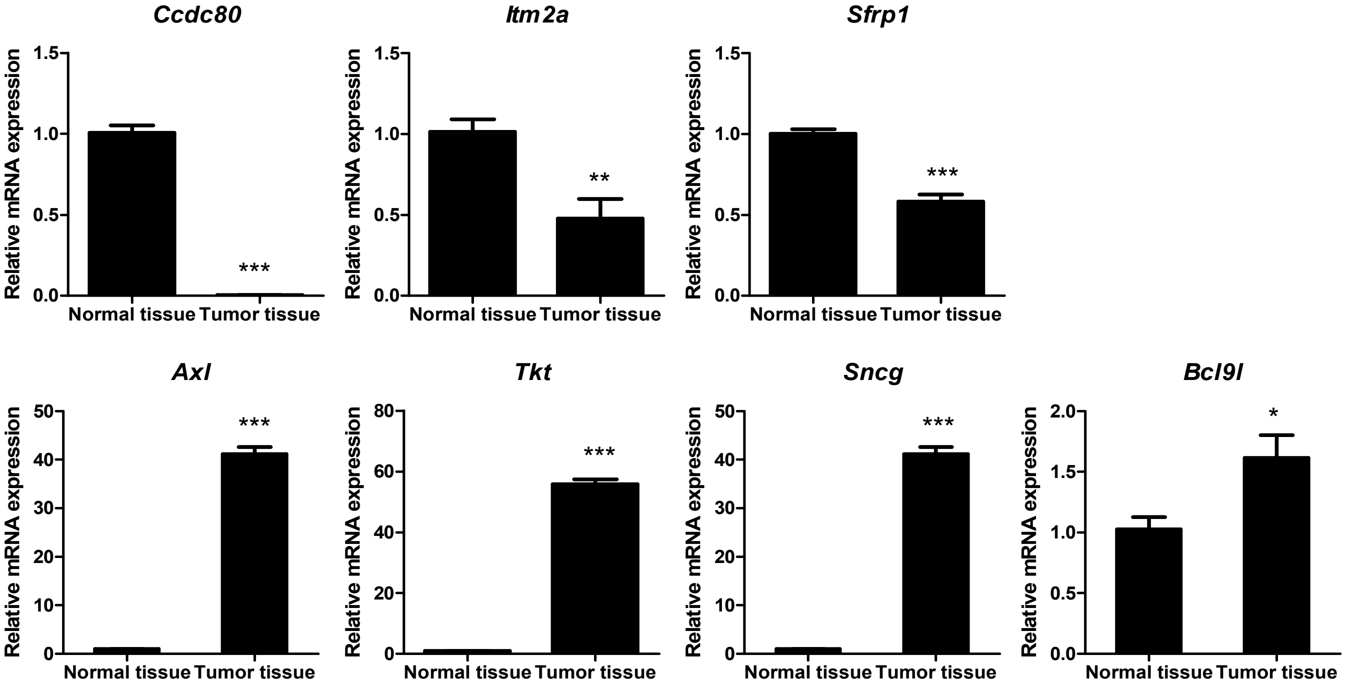

D

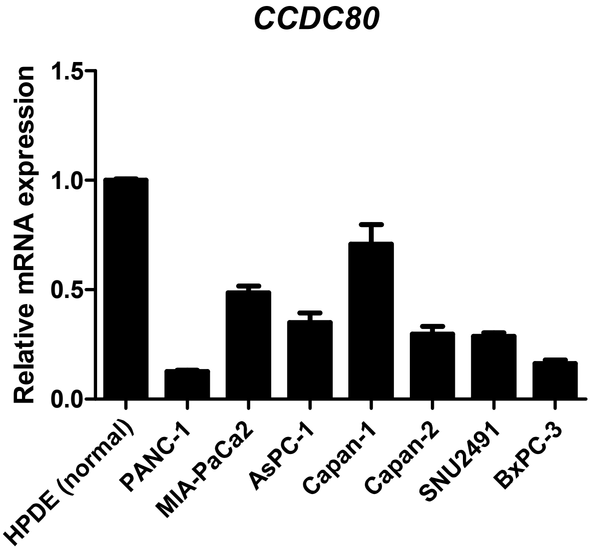

Supplementary figure 6 (continued). FPKM values from RNA sequencing and validation of target genes

(C) Comparing mRNA expression levels of target genes between healthy mouse pancreas and Panc02-induced pancreatic tumor tissue. ***P<0.0005, **P<0.005, and *P<0.05 compared to the control group. (D) Relative mRNA expression of *CCDC80* in human pancreatic cancer cell lines compared to normal pancreatic cell line. Note that tumour-suppressive gene *CCDC80* is down-regulated in mouse pancreatic tumour and human pancreatic cancer cells.

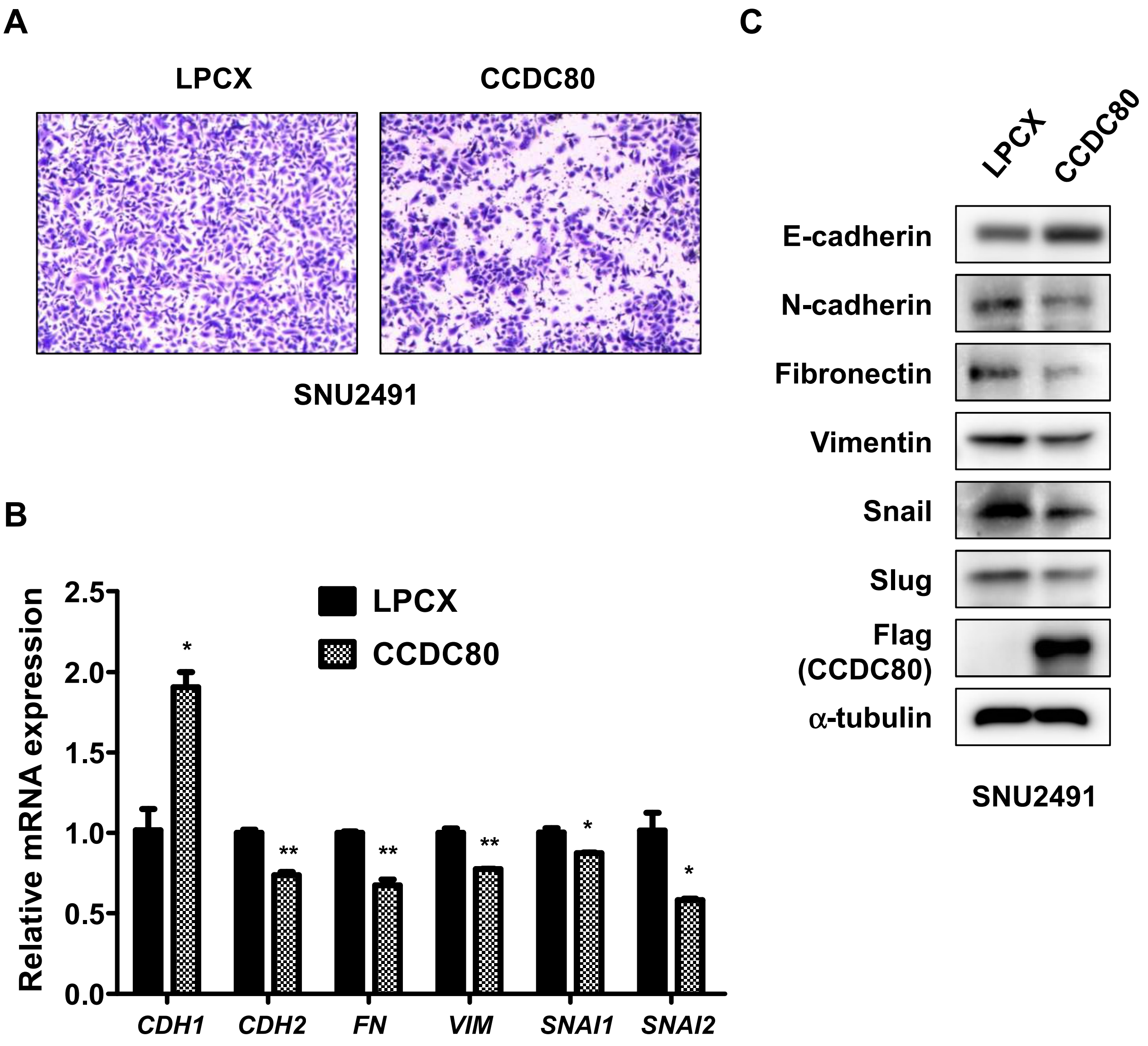

Supplementary figure 7. Reduction of cell migration and EMT markers in SNU2491 stably expressing CCDC80

(A) Transwell migration assay presenting decrease in cell migration by stable expression of CCDC80. (B) Comparing relative mRNA expression levels of EMT markers between LPCX control and CCDC80 expressing cell lines. The values represent the mean ± SD of triplicate data. **P<0.005, and *P<0.05 compared to LPCX. (C) Western blot analysis showing changes in EMT markers in SNU2491 stably expressing CCDC80 compared to the control. The blots are cropped, and the full blots are presented in Supplementary Figure 8.

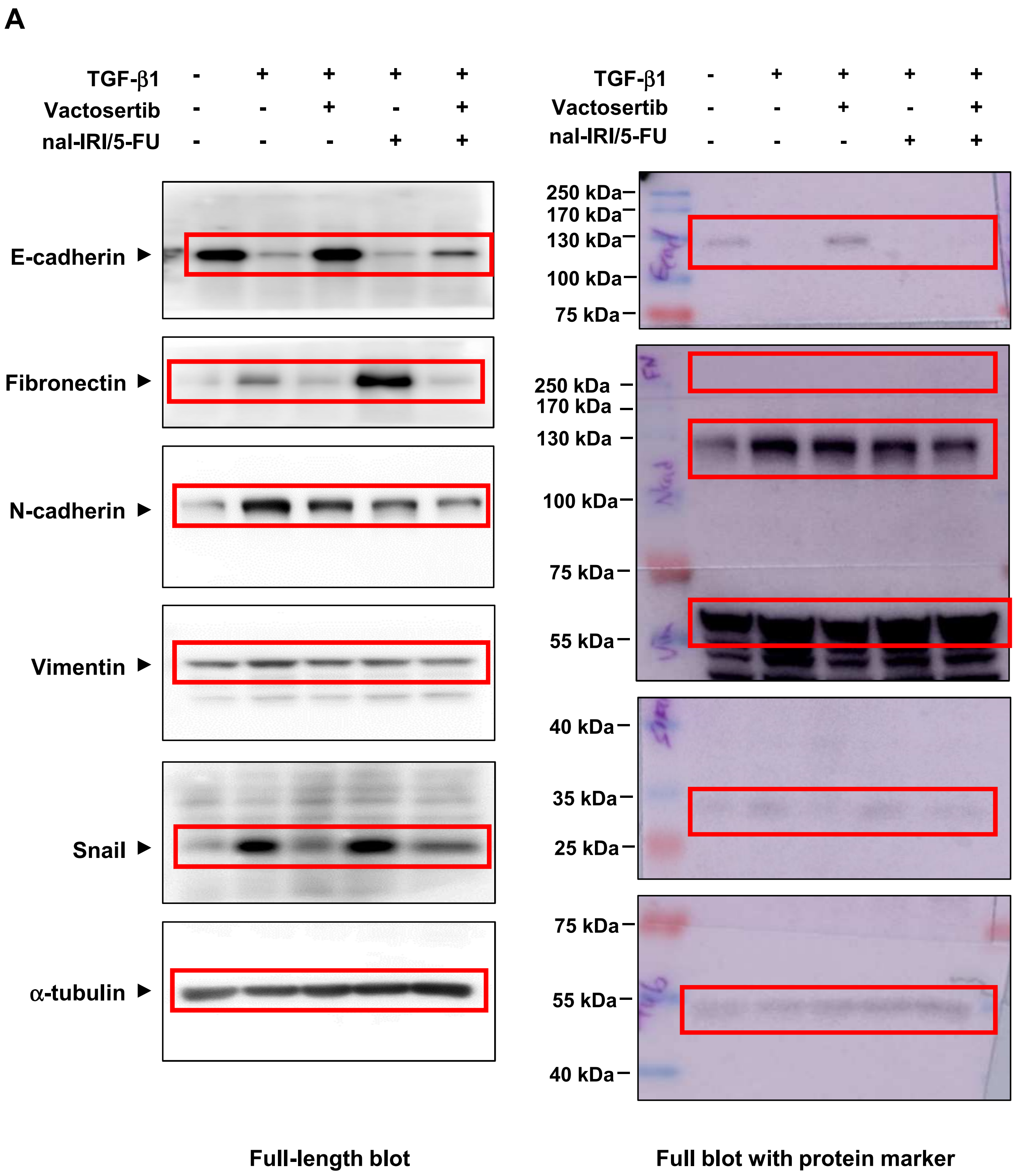

Supplementary figure 8. Full-length blots of cropped images in figures

(A) Full-length blots (left) and full blots with protein marker (right) presenting EMT marker expression of PANC-1 (Figure 2C).

B

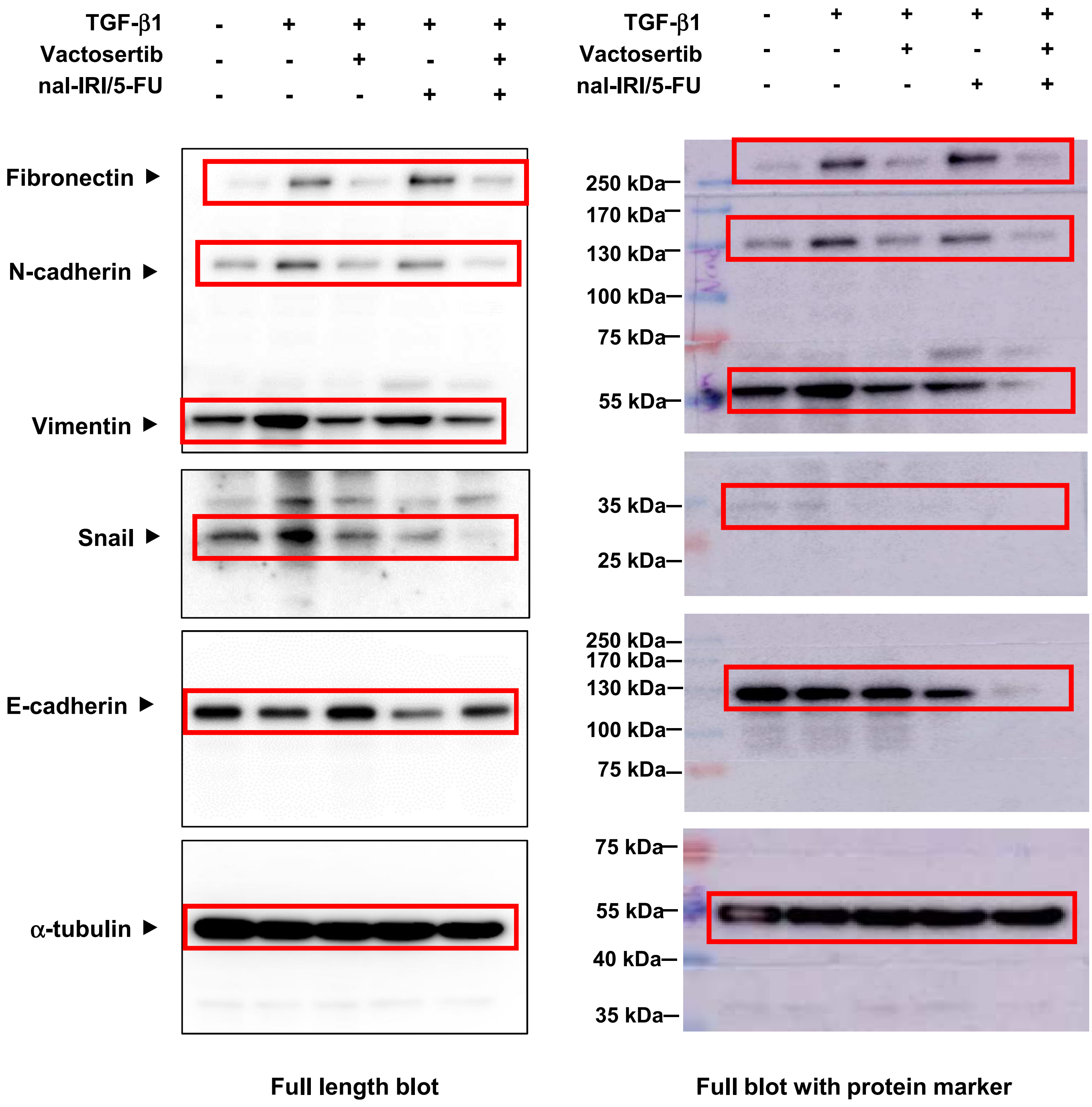

Supplementary figure 8 (continued). Full-length blots of cropped images in figures

(B) Full-length blots (left) and full blots with protein marker (right) presenting EMT marker expression of Panc02 (Figure 2C).

C

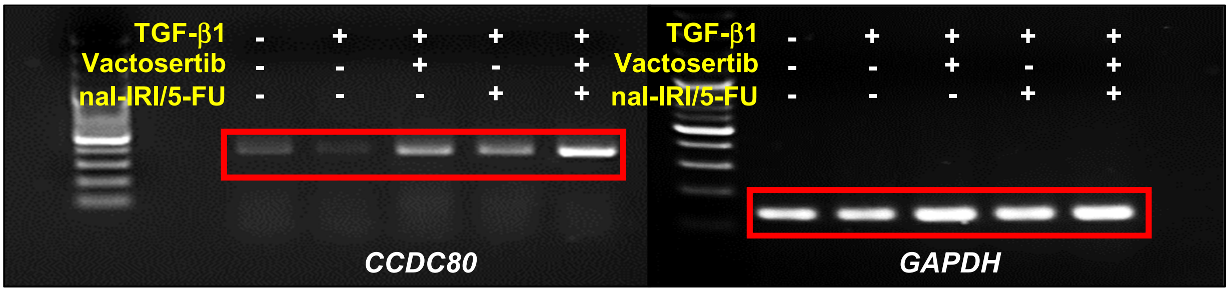

PANC-1

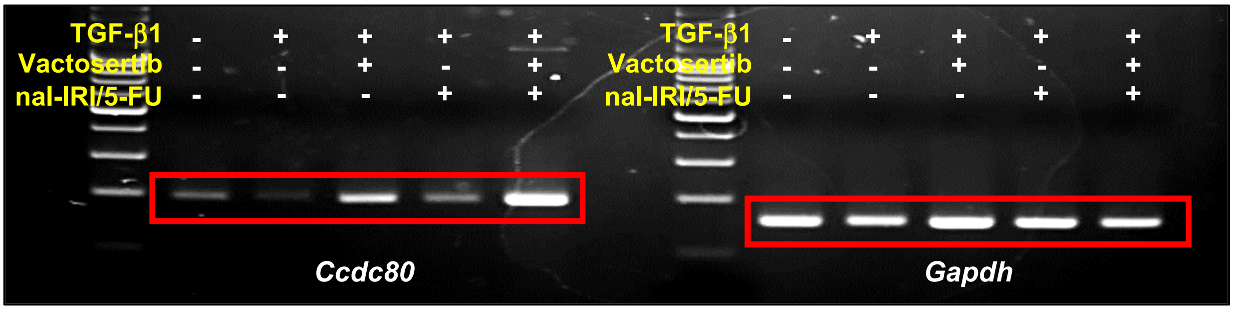

Panc02

Supplementary figure 8 (continued). Full-length blots of cropped images in figures

(C) Full image of RT-PCR presenting *CCDC80* and *GAPDH* expression of PANC-1 (above) and Panc02 (bottom) (Figure 4D).

D

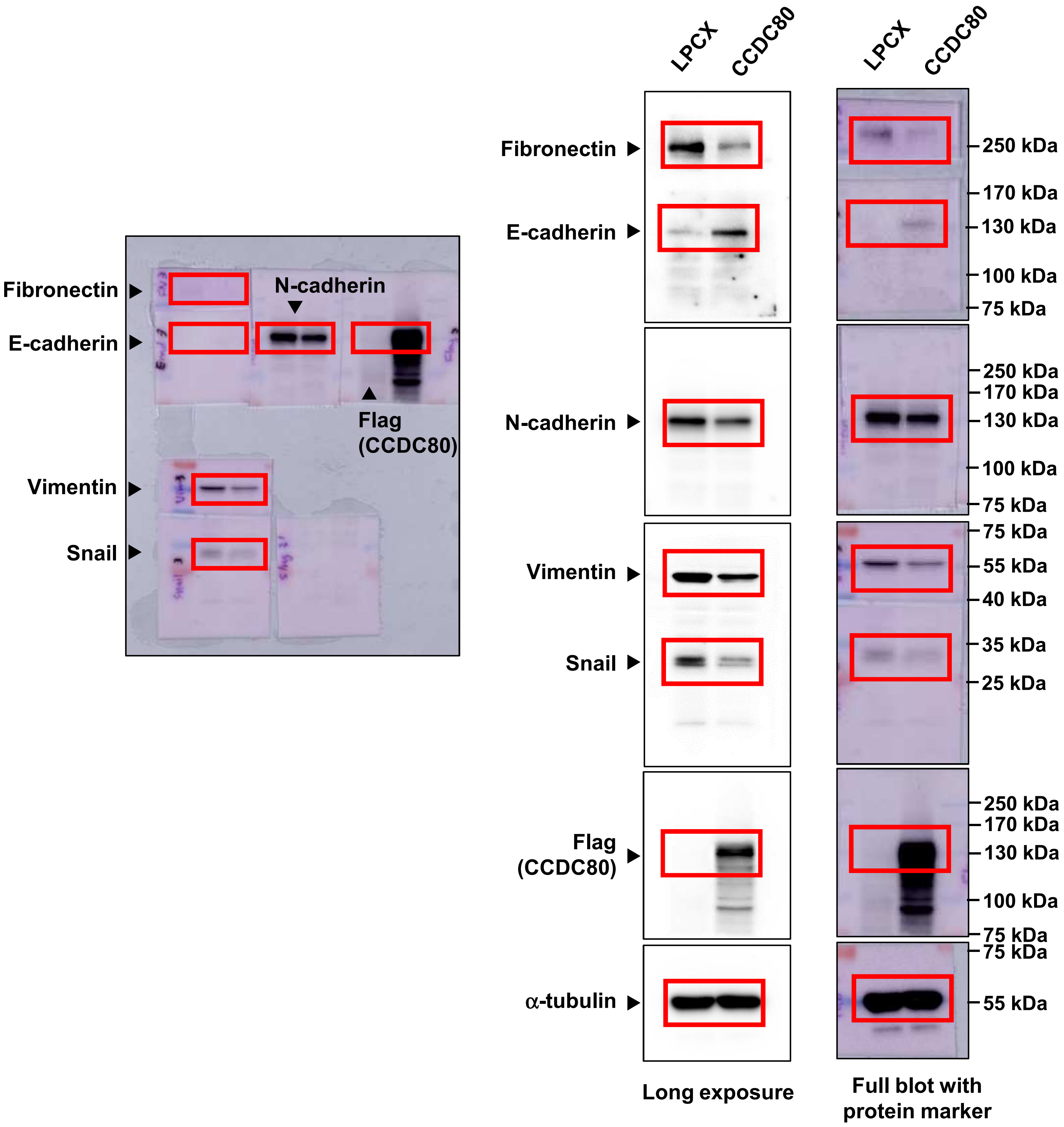

Supplementary figure 8 (continued). Full-length blots of cropped images in figures

(D) Full-length blots and full blots with protein marker presenting EMT marker expression of PANC-1 stably expressing CCDC80 (Figure 5D).

E

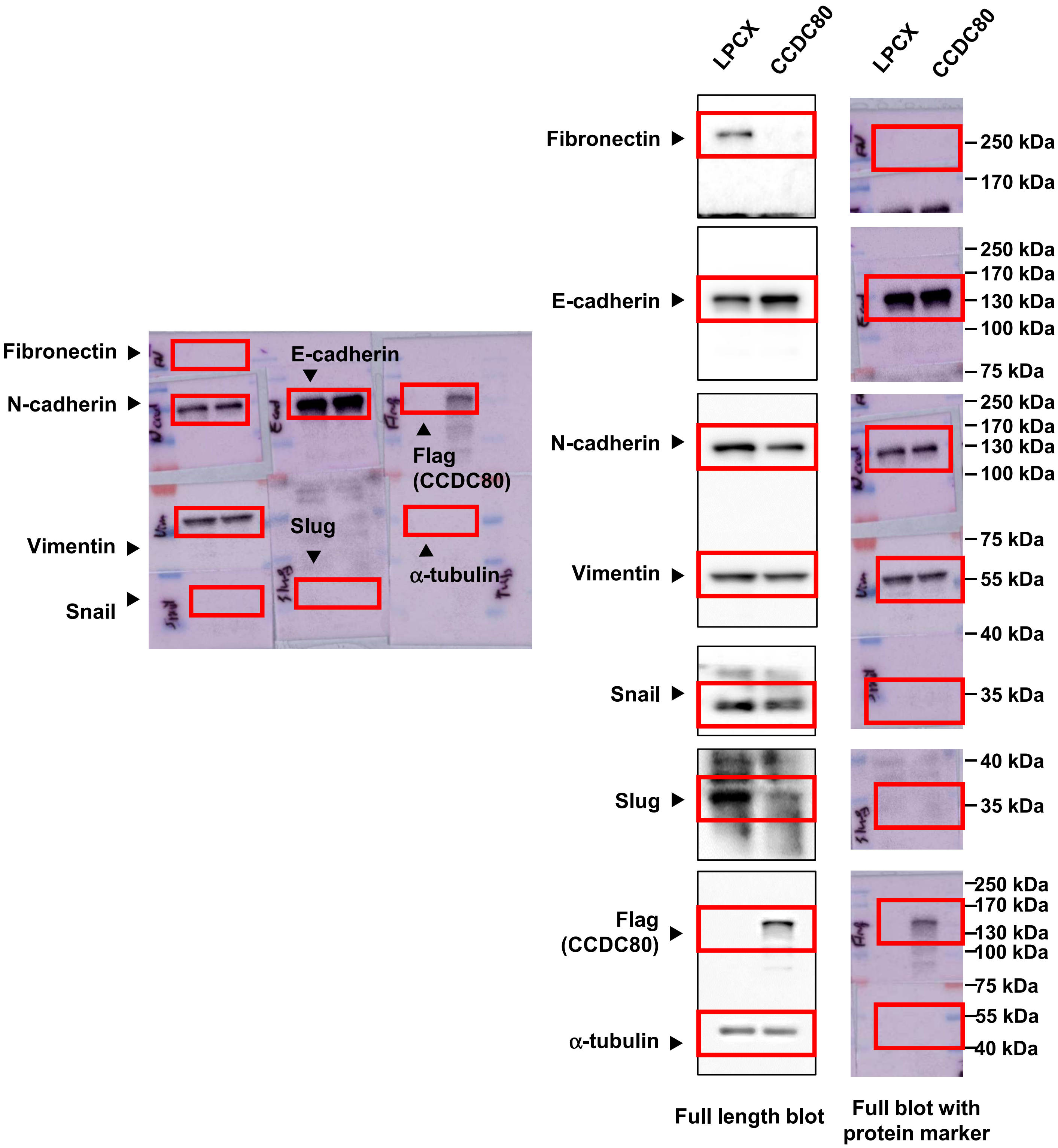

Supplementary figure 8 (continued). Full-length blots of cropped images in figures

(E) Full-length blots and full blots with protein marker presenting EMT marker expression of Panc02 stably expressing CCDC80 (Figure 5D).

F

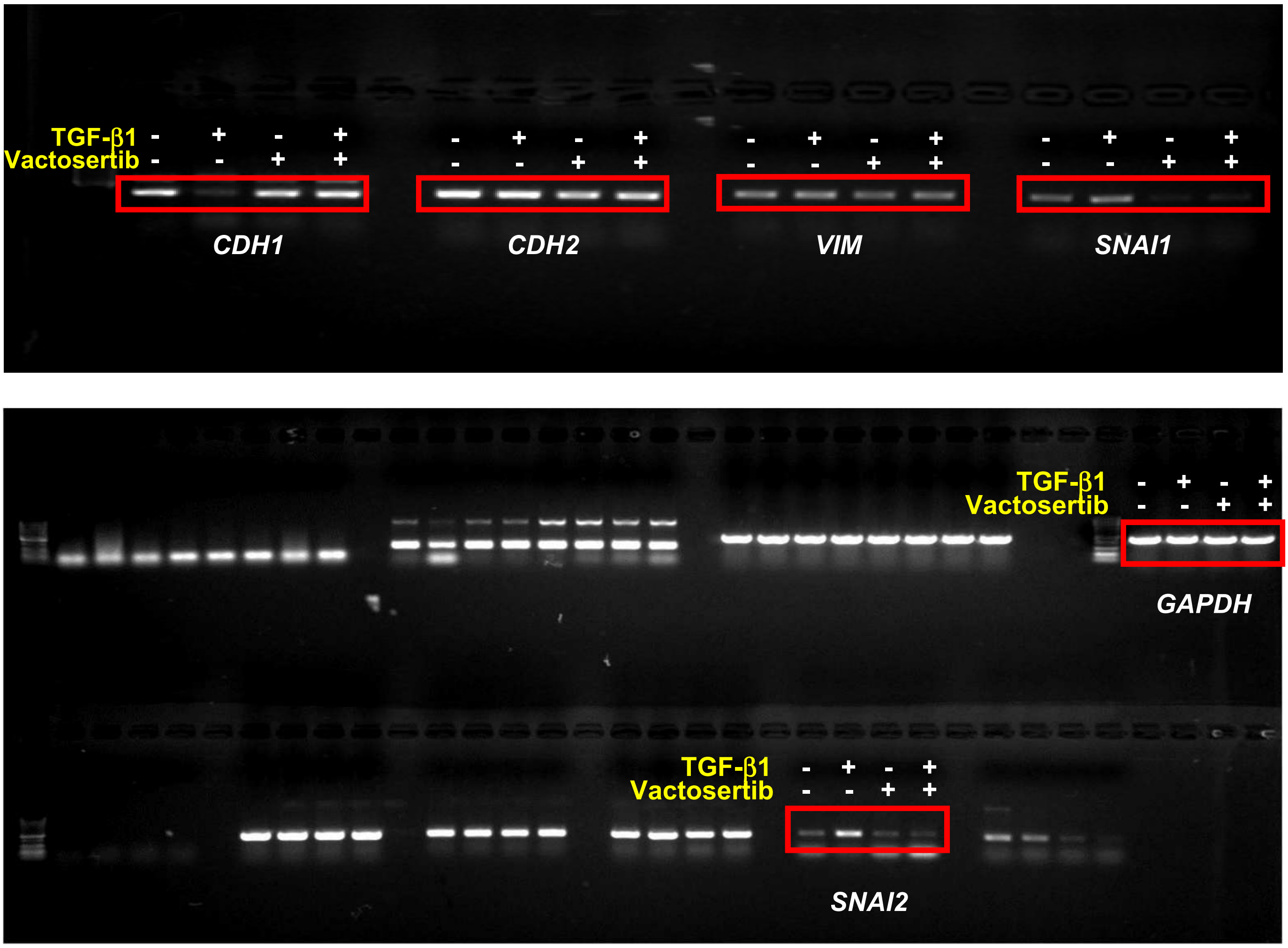

Supplementary figure 8 (continued). Full-length blots of cropped images in figures

(F) Full image of EMT marker RT-PCR in TGF-β1- and vactosertib-treated PANC-1 (Supplementary Figure 1B).

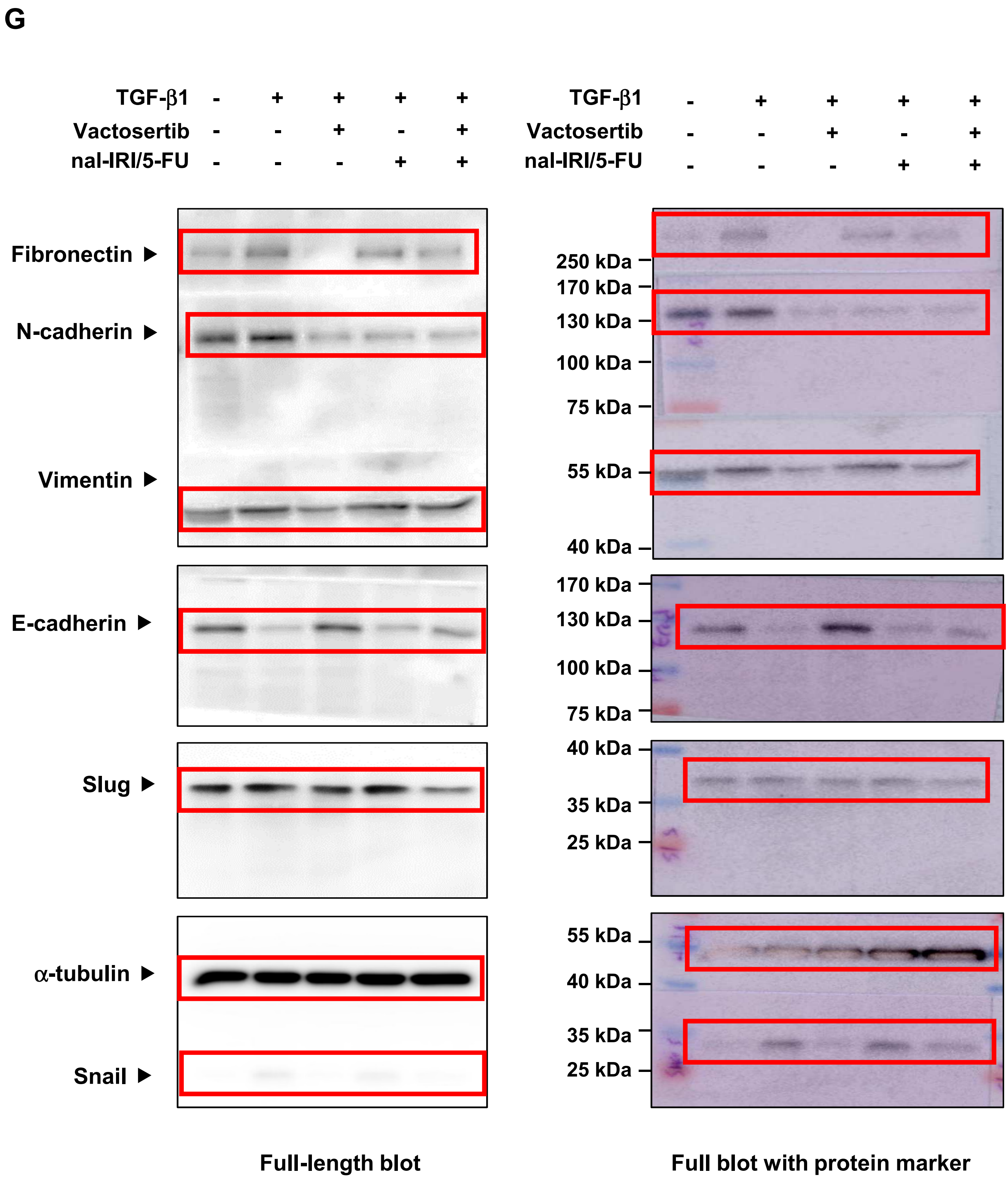

Supplementary figure 8 (continued). Full-length blots of cropped images in figures

(G) Full-length blots (left) and full blots with protein marker (right) presenting EMT marker expression of SNU2491 (Supplementary Figure 5C).

H

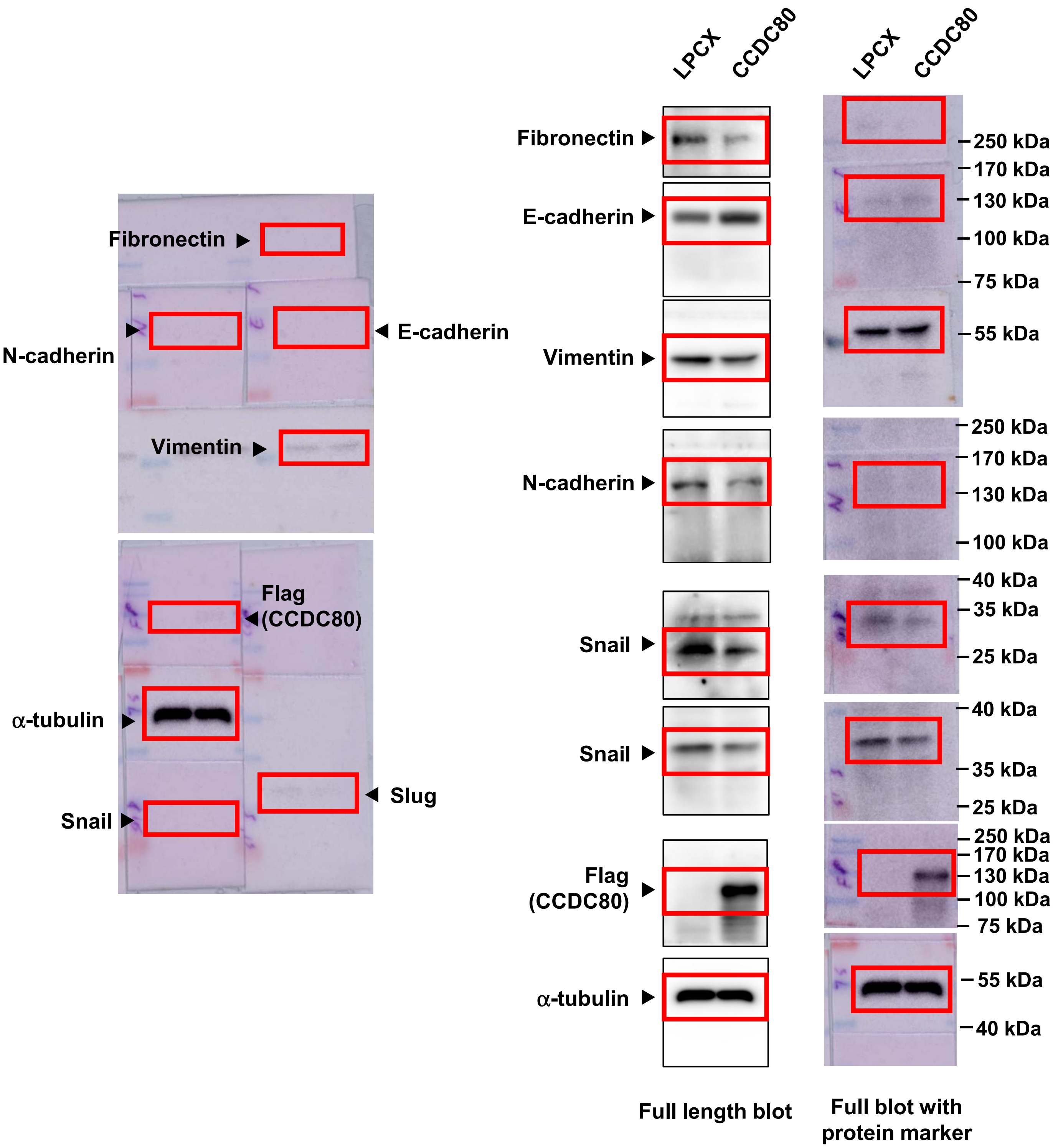

Supplementary figure 8 (continued). Full-length blots of cropped images in figures

(H) Full-length blots and full blots with protein marker presenting EMT marker expression of SNU2491 stably expressing CCDC80 (Supplementary Figure 7C).
